# VTX-PID as a novel recombinant immunoglobulin G–degrading enzyme (IdeS) for efficient AAV-based gene therapy in participants with neutralizing antibodies: results of the phase I first-in-human NAVIgATE study

**DOI:** 10.3389/fimmu.2026.1824802

**Published:** 2026-05-20

**Authors:** Bernard Benichou, Rainard Fuhr, Elodie Vernadal, Sonia Valero, Blanche Tamarit, Veronica Ferrer, Gloria Gonzalez-Aseguinolaza, Anne Douar, Marc Froissart, Annelise Brossel, Simone Floettmann, Francesca Del Bene, Céline Bouquet, Jean-Philippe Combal

**Affiliations:** 1 Vivet Therapeutics SAS, Paris, France; 2 Parexel International GmbH, Berlin, Germany; 3 DNA & RNA Medicine Division, Gene Therapy for Rare Diseases Department, Center for Applied Medical Research (CIMA), University of Navarra, IdisNA, Pamplona, Navarra, Spain; 4 Vivet Therapeutics SL, Pamplona, Spain; 5 Lausanne University Hospital and University of Lausanne, Lausanne, Switzerland; 6 Parexel International SRL, Milan, Italy

**Keywords:** adeno-associated virus, gene therapy, IgG, Imlifidase, neutralizing antibodies, total antibodies, VTX-PID

## Abstract

**Introduction:**

Pre-existing anti-adeno-associated virus (AAV) neutralizing antibodies (NAbs) can impair AAV-mediated gene delivery. VTX-PID (imlifidase) is a streptococcal protease that specifically cleaves immunoglobulin G (IgG) antibodies. NAVIgATE was a first-in-human study of VTX-PID.

**Methods:**

This double-blind controlled study (EUCT number: 2023-503892-83-00) randomized 35 healthy men, stratified by baseline anti-AAV3B NAb levels (intermediate [≤ 1:45] or high [>1:45]) to receive ascending VTX-PID doses (0.075-0.6 mg/kg) or placebo. The primary endpoint was safety. Key secondary endpoints included levels of anti-AAV3B NAbs and total antibodies (TAbs).

**Results:**

VTX-PID resulted in substantial reductions in total IgG, undigested IgG, anti-AAV3B TAb and NAb levels across all doses, followed by gradual full recovery, with effects being sustained longer (2–5 days) at the highest doses. All participants with intermediate NAb levels achieved durable noninhibitory NAb levels (titer ≤1:5), while those with high NAb levels showed reduction but did not reach the noninhibitory level. Most participants experienced VTX-PID-related treatment−emergent adverse events, primarily musculoskeletal complaints of predominately mild to moderate intensity and fully reversible. Transient mild to moderate infusion-associated reactions occurred in four participants, and reversible liver function test elevations in three.

**Discussion:**

VTX-PID effectively depleted anti-AAV3B NAbs, with a dose-dependent effect in magnitude and duration. The safety profile was manageable, with few temporary inflammatory events and self-resolving liver enzyme elevations. VTX-PID 0.3 mg/kg was identified as the appropriate dose based on its safety and efficacy profile. In the population with intermediate NAb levels, the 2- to 5-day depletion in anti-AAV3B NAbs supports VTX−PID as a promising pretreatment approach to open a window of opportunity of up to 5 days, broadening eligibility for systemic AAV-based gene therapies.

## 1 Introduction

Cumulatively, approximately 8000 known genetic disorders affect nearly 10% of the population; however, effective treatments are available for only 5% of these individuals, and most therapies manage symptoms rather than address the underlying cause, resulting in a need for lifelong care (1, 2). In recent years, gene therapy, which aims to provide a functional copy of the defective gene or directly correct genetic mutations through genome editing, has emerged as a promising strategy to treat many of these inherited disorders (1, 2).

Adeno-associated viral (AAV) vectors have become a leading gene delivery platform, demonstrating safety and efficacy across numerous clinical trials for genetic disorders (3, 4). Several recombinant AAV vectors have received regulatory approval for various genetic disorders (1, 2). Additionally, more than 340 AAV-based gene therapy clinical trials are currently registered at (5). However, the seroprevalence of anti-AAV neutralizing antibodies (NAbs), due to either natural AAV exposure or following recombinant AAV gene therapy, can significantly impair the ability of AAVs to deliver therapeutic genes to target cells, thereby preventing their efficacy (6–8). NAbs against AAV vectors are directed against the capsid and are predominantly of the immunoglobulin G1 (IgG1) subclass (9–11). Depending on age, geographical area, ethnic group, and AAV serotype, the inhibitory presence of NAbs affects approximately 38%–75% of patients (7, 9, 12, 13). Patients with inhibitory levels of NAbs, as determined by serological assays, have to date been largely excluded from receiving systemic AAV-based gene therapy (8, 12). Various approaches to cope with anti-AAV antibodies have been tested in animal models; these include plasmapheresis, which proved to be poorly efficient because it necessitates repeated sessions and is generally effective only in animals and patients with low NAbs titers (8, 14, 15). Other proposed methods to reduce anti-AAV antibodies or mitigate AAV-mediated immunotoxicity include enzymatic antibody degradation, glucocorticoid-based immune modulatory regimens, proteasome inhibitors or rituximab for B cell depletion, sirolimus/rapamycin to suppress T and B cell activation, and possible IgG-clearing agents (4, 16). Recent strategies to overcome the effect of NAbs have shown some promise in animal models of passively immunized mice and nonhuman primates (NHPs), but none have proven to be fully effective, especially in NHPs, demonstrating some limitations related to animal model suitability with reduced potency of Ig-degrading protease, originating from *Streptococcus pyogenes* (IdeS) in NHPs (17). Recently, the development of serodivergent nonmammalian AAVs evaded pre-existing antibodies and vector-induced immunity in mice; however, applicability to humans remains very challenging (18). Therefore, there remains a need for a safe and effective solution to mitigate the impact of NAbs in AAV-mediated gene therapy.

VTX-PID (imlifidase per International Nonproprietary Names) is a 35-kDa recombinant form of IdeS produced in *Escherichia coli* that hydrolyzes all four subclasses of human IgG (17). VTX-PID is intended to pretreat patients with pre-existing NAbs against the AAV capsid to obtain their transient elimination to enable efficient AAV−based gene therapy. While VTX-PID is ultimately intended for use with any AAV-based gene therapy, it was initially tested in the clinical setting with a focus on the AAV serotype 3B (AAV3B), as the sponsor is developing AAV3B-based liver gene therapies for Wilson’s disease (EUCT number: 2020-000963-22), progressive familial intrahepatic cholestasis (19, 20), and cerebrotendinous xanthomatosis (21). VTX-PID, whose active substance is imlifidase, refers to the drug developed using the sponsor’s proprietary manufacturing process and formulation. VTX-PID amino acid sequence is identical to that of recombinant imlifidase (IdeS; Idefirix^®^), which has been approved for desensitization treatment of highly sensitized adult kidney transplant patients (22). VTX-PID, like IdeS, acts by sequentially, specifically, and rapidly cleaving both heavy chains of IgG antibodies, first generating a single-cleaved IgG, then cleaving the remaining heavy chain, releasing one F(ab')_2_ fragment and a noncovalently linked homodimeric Fc fragment (23, 24). Preclinical pharmacodynamics (PD) and safety studies in Beagle dogs have indicated that VTX-PID efficiently and dramatically reduces IgG in plasma and is well tolerated (sponsor’s unpublished data [B. Tamarit, 2023]). Other preclinical studies have shown that IdeS is highly specific and efficient at cleaving human IgG and that preconditioning with IdeS positively affects AAV3B-mediated liver transduction in the presence of anti-AAV3B NAbs (17).

The phase I first-in-human randomized NAVIgATE (NAb AAV IgG Trial with Endopeptidase) study was conducted to evaluate VTX-PID in healthy male participants stratified by intermediate or high baseline anti-AAV3B NAb levels. Here we report safety, PD, pharmacokinetics (PK), and immunogenicity outcomes from NAVIgATE.

## 2 Materials and methods

### 2.1 Participants

Eligible participants were healthy males aged 18–55 years, with body mass index between 19 and 29.9 kg/m^2^, who present with intermediate or high anti-AAV3B NAb levels at screening. Participants were fully or sufficiently vaccinated against severe acute respiratory syndrome coronavirus 2 (SARS-CoV-2), smoked five or fewer cigarettes per day, and agreed to abstain from using any nicotine-containing products starting ≥24 hours prior to screening through their enrollment. Additionally, enrolled participants gave written informed consent and agreed to use effective pregnancy prevention with their partners of childbearing potential. Exclusion criteria included clinically significant immunodeficiency, inadequate tetanus or pneumococcal antibody levels, estimated glomerular filtration rate <90 mL/min/1.73 m^2^, notable disease or recent illness, abnormal laboratory results, and chronic infections. Other exclusion criteria included positive tests for hepatitis B/C or HIV, SARS−CoV−2, or symptoms of active herpes, recent live vaccinations, history of substance abuse, recent blood donation, hypersensitivity to study treatments, concurrent study enrollment, or prior severe COVID−19 requiring hospitalization.

### 2.2 Study design and treatment

NAVIgATE (EUCT number: 2023-503892-83-00) was a phase I, single-dose, double-blind, randomized, placebo-controlled first-in-human clinical trial that enrolled participants at a single center in Germany. Eligible participants were randomized in a 3:1 ratio to either VTX-PID treatment or placebo across four cohorts with escalating doses. The four VTX-PID dose levels administered at Day 1 of the study schedule were 0.075 mg/kg (Cohort 1), 0.15 mg/kg (Cohort 2), 0.3 mg/kg (Cohort 3), and 0.6 mg/kg (Cohort 4). The dose escalation per consecutive cohorts design of the study ensured participant safety. Eligible participants were stratified by baseline circulating anti-AAV3B NAb levels (intermediate or high). Accordingly, an anti-AAV3B NAb assay was developed and validated in accordance with International Council for Harmonisation of Technical Requirements for Pharmaceuticals for Human Use (ICH) guidelines to define three NAb thresholds to rank individuals as negative (titer <1:5, i.e., the noninhibitory NAb level), intermediate (1:5≤ titer <1:45), or high (titer ≥1:45). This stratification also ensured representation of the population most likely to benefit from VTX-PID, i.e., those currently excluded from AAV−based gene therapies due to inhibitory NAb levels. The sponsor determined the threshold based on the assumption that if NAb reduction is proportional to IgG reduction, an eight- to ten-fold decrease in NAbs could be expected upon VTX-PID treatment. NAb titers were determined as the reciprocal dilution of serum using an ICH guideline–validated cell-based transduction inhibition assay’s cut point. Considering the dilution ratio and screening criteria, samples were considered negative whenever transduction efficiency was superior to the cut point at a dilution of 1:5 (25); the intermediate threshold was defined as <1:45, which corresponds to approximately 55% of the NAb-positive population or about 19% in absolute number of AAV3B population (considering both seronegative and seropositive population). Each cohort was designed to include eight randomized participants, stratified by anti-AAV3B NAb levels, when feasible, with four participants having intermediate levels and four having high levels (three receiving VTX-PID and one receiving placebo at each NAb level). For each cohort, a sentinel group of two participants (one receiving VTX-PID and one receiving placebo) was dosed first. The sentinel groups were assessed to determine continued enrollment in the cohort.

Three additional nonrandomized participants in the intermediate NAb level subgroup were planned to receive either 0.6 mg/kg VTX-PID or the Safety Review Committee–selected dose after completion of the dose escalation phase (Day 14 following the last participant in Cohort 4). Following a review of safety data from the 0.6 mg/kg VTX−PID cohort, the Safety Review Committee decided to administer the lower dose of 0.3 mg/kg to the nonrandomized participants instead. The inclusion of the three additional participants, who underwent out-of-sequence dosing, aimed to provide more safety data at the dose of 0.3 mg/kg VTX-PID in participants receiving prophylactic corticosteroids and to consolidate data for various anti-AAV3B NAb levels.

Participants received VTX-PID or placebo (saline solution) as a single-dose intravenous (IV) infusion over 30 minutes at 0.075, 0.15, and 0.3 mg/kg, and 60 minutes for the 0.6 mg/kg dose level, on Day 1. Safety assessments were conducted before, at, and, after dosing. After treatment administration, safety monitoring and serial blood sampling for PD evaluation were performed throughout the hospitalization period from Day 1 up to Day 14. Prophylactic premedication included oral antihistamine (loratadine 10 mg, 1 hour before infusion) and oral antibiotic (amoxicillin 3 × 500 mg daily or other permitted antibiotics with similar activity on encapsulated bacteria) from Day 1 before study intervention infusion until Day 28 or earlier if total serum IgG levels restored to ≥4.5 g/L. Oral corticosteroid therapy of prednisolone 60 mg ~1.5 hours before infusion on Day 1, and 30 mg 24 hours post VTX-PID infusion, was incorporated into the treatment regimen for three participants in Cohort 4 and the three nonrandomized participants; this therapy was implemented to mitigate potential infusion-associated reactions and VTX-PID–related treatment-emergent adverse events (TEAEs). From Day 7 onward, participant discharge was based on daily assessments of total IgG level recovery. The study lasted up to 120 days for each participant, and included a screening period (up to 28 days), study center confinement from Day –1 up to Day 14 (minimum of 7 days), with treatment administration on Day 1, and outpatient visits until Day 90 (end of the study). When the final participant at a given dose level completed the 14-day follow-up (Visit 4), the Safety Review Committee was unblinded and reviewed safety, PD, and PK data to recommend dose escalation or continued dosing. The study design included predefined stopping criteria, which, if met, would result in temporary suspension or termination of the clinical trial.

### 2.3 Objectives and endpoints

The primary objective was the evaluation of safety and tolerability of single ascending IV infusions of VTX-PID. The primary endpoint was safety after a single VTX-PID IV infusion. A secondary endpoint was PD as assessed by levels of anti-AAV3B NAbs and anti-AAV3B total antibodies (TAbs) over time and circulating concentrations of total IgG, undigested IgG and F(ab')_2_ fragments (from IgG1, IgG3, and IgG4) after single ascending IV infusions of VTX-PID. Other secondary endpoints included characterization of VTX-PID plasma PK parameters and of the humoral immune response to VTX-PID by monitoring anti-VTX-PID antidrug antibody (ADA) levels over time.

### 2.4 Assessments

Safety was based on constant monitoring of AEs and their clinical assessments, vital signs, weight, 12-lead electrocardiograms, cardiac telemetry, clinical safety laboratory tests, and physical examination. TEAEs were defined as those that first appeared or worsened in severity following administration of the study drug. AEs were graded as mild, moderate, or severe per protocol definition (common scale) and the causality was assessed by the investigator. Reported AE terms were coded using the Medical Dictionary for Regulatory Activities (MedDRA) v28.0. AEs of special interest included infusion-related reactions that occurred less than 24 hours after VTX-PID administration, severe or serious infections post VTX-PID through end of study, and lack of trend for IgG recovery by Day 14. All AEs were collected from the screening visit (Visit 1), following signed informed consent, until the end of study/early termination visit (Visit 6) at prespecified time points.

Blood samples of approximately 30 mL were collected and processed at prespecified time points for evaluation of PK of VTX-PID in plasma using a validated assay, which measured VTX-PID signature peptide GGIFDAVFTR by liquid chromatography with tandem mass spectrometric detection.

For PD analyses, samples were collected at designated intervals to assess anti-AAV3B TAbs and circulating anti-AAV3B NAbs and to quantitatively analyze total and undigested IgG levels and serum F(ab')_2_ fragments from IgG hydrolyzation. Anti-AAV3B NAb levels were evaluated using a validated cell-based assay, with a negative threshold titer of <1:5. Anti-AAV3B TAb levels were determined using a validated electrochemiluminescent Meso Scale Discovery–based assay. Total IgG levels were measured via a turbidimetry assay. Total undigested IgG levels were determined using a validated Meso Scale Discovery–based assay that quantified intact, undigested IgG molecules exclusively and did not detect cleaved fragments such as F(ab')_2_; this distinction allowed for precise monitoring of the enzymatic activity of VTX−PID by measuring the reduction in intact IgG and the corresponding increase in cleavage products over time. The levels of F(ab')_2_ fragments were analyzed in stabilized (with iodoacetic acid) serum samples after *in vitro* tryptic digestion using a qualified liquid chromatography-mass spectrometry/mass spectrometry method that consisted of determining the concentration of three signature peptides, THTCPPCPAPELLG, CPAPELLG, and YGPPCPSCPAPEFLG, representative of IgG1, IgG3, and IgG4, respectively.

Immunogenicity was assessed by measuring anti-VTX-PID–binding ADAs in serum samples at predefined time points, according to a tiered approach (screening/confirmation/titer), using a validated electrochemiluminescence assay after anti-VTX-PID magnetic bead immunoprecipitation. Titers were reported for confirmed positive samples. Regarding treatment-boosted immunogenicity considerations, a significant increase in the postdose sample titer, i.e., when the fold-increase was greater than the minimum significant ratio of the assay (MSR = 3.674), was considered indicative of a treatment-boosted ADA response.

### 2.5 Ethics statement

This study was conducted in accordance with the protocol and consensus ethical principles derived from the Declaration of Helsinki (Version 2013), Council for International Organizations of Medical Sciences International Ethical Guidelines (26), applicable ICH Good Clinical Practice Guidelines, applicable ISO 14155 medical device guidelines, as well as the requirements of the European Union Data Protection Directive 95/46/EC, and other applicable regulatory requirements. All participants signed written informed consent before undergoing any study-related procedures.

### 2.6 Statistical analysis

The total sample size for the trial was 35 healthy male participants. Of these, 32 were randomized, eight per cohort, and three additional participants were nonrandomized. No formal power calculations were performed. Safety was assessed in the safety population dataset, which included all participants who received at least one full or partial IV infusion of VTX-PID or placebo. The PK population dataset included all randomized participants who received placebo or VTX-PID and had sufficient samples to determine at least one evaluable PK parameter after a single IV infusion of VTX-PID or placebo, without important protocol deviations affecting the PK variables. Single-dose PK parameters (maximum observed plasma concentration [C_max_] and area under the concentration−time curve [AUC]) were evaluated for dose proportionality by means of power model using the linear regression method. PK data were summarized by descriptive statistics, and the individual participant concentration-time data were displayed graphically on the linear and log scales. The PD population dataset included all randomized participants who received one dose of study drug and had at least one postbaseline PD parameter value available. Continuous data were summarized by treatment using descriptive statistics when number of contributing participants was greater than two, in terms of mean, standard deviation (SD), median, minimum, maximum, and number of observations. Categorical data were summarized by cohort using frequency tables (number and percentage). For AEs, Cohorts 3 and 4 were further categorized based on the administration of corticosteroids.

## 3 Results

### 3.1 Participants

Between October 2023 and June 2025, a total of 35 participants were included in the study and dosed: i) 32 participants were randomized across four dose cohorts (0.075, 0.15, 0.3, and 0.6 mg/kg) and the pooled placebo group (**Supplementary Figure 1A**); all randomized participants received the assigned dose and completed the study; ii) thereafter, three additional nonrandomized participants received VTX−PID 0.3 mg/kg after the escalation phase. Each cohort comprised six participants treated with escalating doses of VTX-PID and two treated with the placebo; Cohort 3 comprised nine participants treated with VTX-PID (**Supplementary Figure 1B**). All participants completed the study treatment. The overall median age across all participants was 39 years (range, 26–55). Most participants were White (97.1%). The overall median body mass index was 24.7 kg/m^2^ (range, 19.1–29.6). The demographics and baseline characteristics were balanced across the four cohorts and pooled placebo group (**Table 1**).

**Table 1:** Baseline demographics of participants across the four cohorts and pooled placebo group.

Characteristic	Cohort 1 VTX-PID (0.075 mg/kg) (*n* = 6)	Cohort 2 VTX-PID (0.15 mg/kg) (*n* = 6)	Cohort 3 VTX-PID (0.3 mg/kg) (*n* = 9)*	Cohort 4 VTX-PID (0.6 mg/kg) (*n* = 6)	Pooled placebo (*n* = 8)
Age, years, median (range)	41.0 (35–52)	41.0 (32–46)	38.0 (28–55)	39.0 (27–45)	37.5 (26–55)
Race, *n* (%)					
White	6 (100)	6 (100)	9 (100)	6 (100)	7 (87.5)
Native Hawaiian or Other Pacific Islander	0	0	0	0	1 (12.5)
Weight, kg, median (range)	73.7 (65.9–85.7)	89.6 (71.5–96.9)	86.5 (66.2–98.7)	77.2 (66.4–83.6)	77.2 (53.3–94.6)
BMI, kg/m^2^, median (range)	23.7 (20.8–25.3)	26.5 (23.3–29.0)	28.2 (21.1–29.6)	23.4 (22.3–27.0)	25.3 (19.1–29.0)

*Three nonrandomized participants were included and treated with 0.3 mg/kg VTX-PID (Cohort 3) after the end of the escalation phase (Day 14 of the last participant for Cohort 4) along with randomized participants.

### 3.2 Safety

All 35 treated participants were included in safety analysis set. All participants in Cohorts 1 and 4, 50.0% in Cohort 2, 88.9% in Cohort 3, and 50.0% in pooled placebo group reported at least one TEAE, as presented in **Supplementary Tables S1**, **S2**. Overall, most participants experienced mild (71.4%) or moderate (48.6%) TEAEs, while TEAEs considered severe were reported in 5.7% of participants (**Supplementary Table 1**). Notably, no serious TEAEs leading to study discontinuation or dose modifications were reported during the study. Additionally, no dose-limiting toxicity or deaths occurred.

Overall, TEAEs deemed related to VTX−PID were reported in 50.0%, 50.0%, 77.8%, and 100% of participants in Cohorts 1, 2, 3, and 4, respectively (**Table 2**). One participant (12.5%) in the pooled placebo group reported treatment-related TEAEs. The most common treatment-related TEAEs reported with VTX-PID were predominately musculoskeletal complaints, including myalgia (34.3%) and arthralgia (25.7%), as well as C-reactive protein increase (17.1%) and pyrexia (17.1%). These related TEAEs were mostly noted at the highest doses (Cohorts 3 and 4) (**Table 2**). Mild to moderate infusion-related reaction was reported in one participant (11.1%) in the Cohort 3/with-corticosteroid subgroup and three (50.0%) in the Cohort 4/without-corticosteroid subgroup. These infusion-related reactions occurred approximately 4–5 minutes after infusion initiation and resolved spontaneously within 4 hours without intervention. Notably, most of them occurred in the without-corticosteroid group of Cohort 4, suggesting a potential protective effect of corticosteroid administration against infusion reactions. Dose-dependent transient elevations in high-sensitivity C-reactive protein were observed across VTX−PID cohorts that typically resolved within 2 weeks.

**Table 2:** Related TEAEs across the four cohorts and pooled placebo group.

Participants with event, *n* (%)	Cohort 1 VTX-PID (0.075 mg/kg) (*n* = 6)	Cohort 2 VTX-PID (0.15 mg/kg) (*n* = 6)	Cohort 3 VTX-PID (0.3 mg/kg) (*n* = 9)*			Cohort 4 VTX-PID (0.6 mg/kg) (*n* = 6)			Pooled placebo (*n* = 8)
			Without cortico-steroid (*n* = 6)	With cortico-steroid (*n* = 3)	Overall	Without cortico-steroid (*n* = 3)	With cortico-steroid (*n* = 3)	Overall	
Any related TEAE^†^	3 (50.0)	3 (50.0)	4 (44.4)	3 (33.3)	7 (77.8)	4 (66.7)	2 (33.3)	6 (100.0)	1 (12.5)
Most frequent related TEAEs^‡^									
Myalgia	3 (50.0)	2 (33.3)	1 (11.1)	2 (22.2)	3 (33.3)	2 (33.3)	2 (33.3)	4 (66.7)	0
Arthralgia	0	1 (16.7)	3 (33.3)	2 (22.2)	5 (55.6)	2 (33.3)	1 (16.7)	3 (50.0)	0
C-reactive protein increased	0	0	3 (33.3)	0	3 (33.3)	2 (33.3)	1 (16.7)	3 (50.0)	0
Pyrexia	0	0	3 (33.3)	0	3 (33.3)	2 (33.3)	1 (16.7)	3 (50.0)	0
Abdominal discomfort	0	0	2 (22.2)	0	2 (22.2)	1 (16.7)	1 (16.7)	2 (33.3)	0
Infusion-related reaction	0	0	0	1 (11.1)	1 (11.1)	3 (50.0)	0	3 (50.0)	0
Lymphadenopathy	0	0	0	1 (11.1)	1 (11.1)	2 (33.3)	0	2 (33.3)	0
Alanine aminotransferase increased	0	1 (16.7)	0	0	0	1 (16.7)	0	1 (16.7)	0
Aspartate aminotransferase increased	0	1 (16.7)	0	0	0	1 (16.7)	0	1 (16.7)	0
Dizziness	0	0	1 (11.1)	0	1 (11.1)	1 (16.7)	0	1 (16.7)	0
Fatigue	0	0	0	1 (11.1)	1 (11.1)	0	0	0	1 (12.5)
Pain in jaw	0	1 (16.7)	1 (11.1)	0	1 (11.1)	0	0	0	0
Rash	0	0	1 (11.1)	0	1 (11.1)	1 (16.7)	0	1 (16.7)	0
Sleep disorder	0	0	0	0	0	0	2 (33.3)	2 (33.3)	0

*Three nonrandomized participants were included and treated with 0.3 mg/kg VTX-PID (Cohort 3) after the end of the escalation phase (Day 14 of the last participant for Cohort 4) along with randomized participants. ^†^Related TEAEs includes both “TEAE only related to study drug” and “TEAE related to study drug combined with other trial drug.” ^‡^Related TEAEs by preferred term are listed if they were reported in ≥2 participants overall. TEAE, treatment-emergent adverse event.

Transient elevations in alanine aminotransferase (ALT) were observed in several participants across different VTX-PID dose cohorts, with a pattern suggesting variable inter−participant sensitivity and the most frequent and prominent increases occurring at the highest dose level of 0.6 mg/kg. Generally, in Cohorts 1–3, ALT levels remained near the normal range (0–50 IU/L). One participant in Cohort 2 experienced an asymptomatic ALT and aspartate aminotransferase (AST) elevation, peaking 7 days post dose (ALT, ~5.6 × upper limit of normal [ULN], which self-resolved within 6 days, and AST, 123.8 IU/L), and one in Cohort 3 had a mild and short ALT increase 6 days post dose (did not exceed 2 × ULN). Cohort 4 had the most pronounced ALT elevations, with two participants experiencing clinically significant increases. One participant exhibited ALT levels peaking at 306.5 IU/L (6.2 × ULN) 5 days post dose, which subsequently decreased by 8 days post dose; the participant also showed significant elevations in other liver function enzymes, AST (3.5 × ULN) and alkaline phosphatase ([ALP], 1.8 × ULN), which met predefined stopping criteria, resulting in temporary suspension of the clinical trial. This prompted the implementation of the prophylactic regimen utilizing concomitant corticosteroid therapy of prednisolone. The other participant demonstrated the highest ALT elevation in the study, reaching 696.2 IU/L (~14 × ULN) 6 days post dose, which decreased by 8 days post dose; this participant also had elevations in AST (6.4 × ULN) and ALP (1.2 × ULN). Both these participants experienced transient Common Terminology Criteria for Adverse Events (CTCAE) grade 3 ALT elevations that resolved without specific intervention. Participants in the pooled placebo group maintained stable ALT values within the normal range, supporting the conclusion that the observed ALT elevations with VTX-PID were treatment related and predominately observed at the highest dose of 0.6 mg/kg.

No clinically significant changes in hematology, vital signs, physical examinations, 12-lead electrocardiograms, or coagulation were identified, except as noted. One participant in the Cohort 4/without-corticosteroid subgroup had clinically significant temperature of 39.6 °C 9 days post dose. Transient proteinuria (mean range, 0.342–0.767 g/L), identified by urinalysis, was observed 24–48 hours after dosage of VTX-PID in all cohorts, most notably at the two highest doses administered; urine protein levels largely returned to normal by 3 days post dose.

### 3.3 Pharmacokinetics

**Figure 1:**
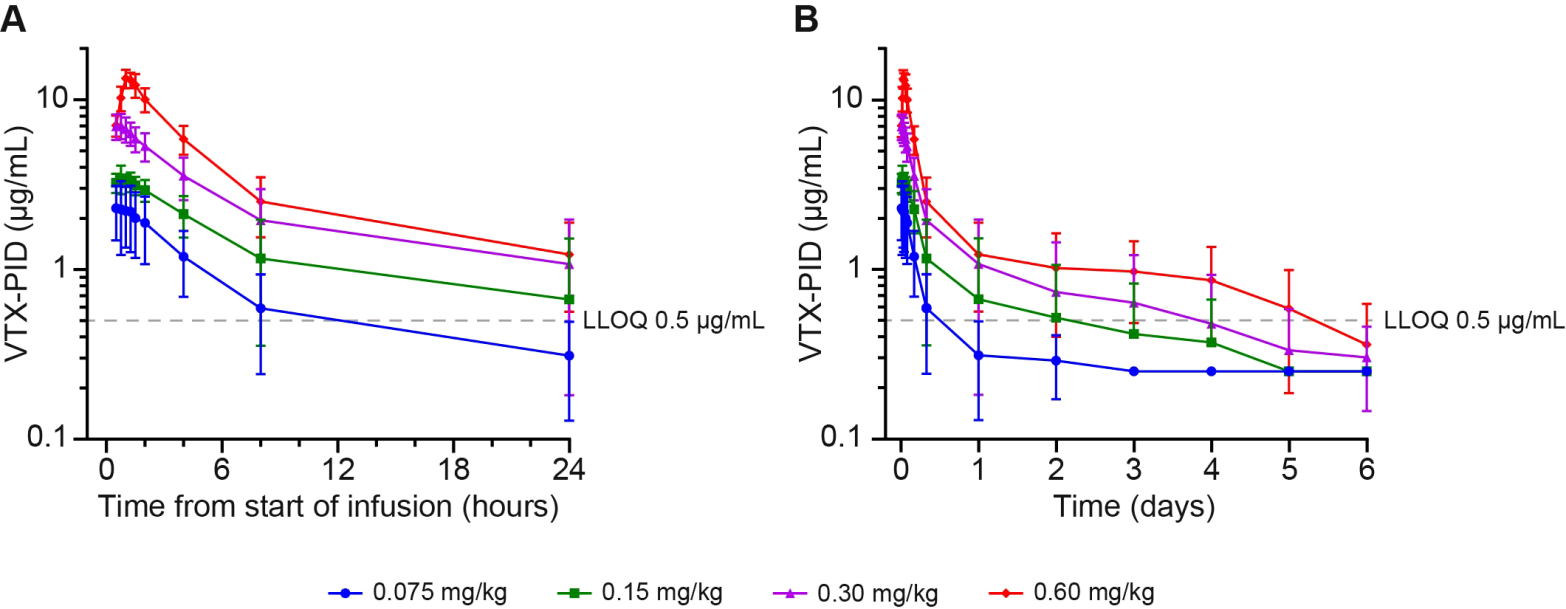
Arithmetic mean (± SD) VTX-PID plasma concentration during **(A)** the first 24 hours and **(B)** up to 6 days post dose across all four cohorts. The dashed gray line represents the method LLOQ at 0.5 µg/mL. When result below the limit of quantification was obtained, a value of LLOQ value/2, i.e., 0. 25 µg/mL, was used for representation. LLOQ, lower limit of quantification; SD, standard deviation.

All cohorts exhibited rapid initial distribution in plasma following intravenous administration of VTX-PID, with peak concentrations observed at a median time to C_max_ of 0.75–0.78 hours after start of infusion for Cohorts 1–3 and 1.13 hours for Cohort 4, followed by a multiphasic decline (**Figure 1A**). The 0.3 mg/kg and 0.6 mg/kg doses maintained quantifiable concentrations (above the lower limit of quantification [LLOQ] of 0.5 µg/mL) throughout the entire 144-hour sampling period, while lower-dose cohorts approached or fell below quantification limits earlier (**Figure 1B**). Pre-existing ADAs appeared to influence VTX-PID PK across all dose cohorts, with participants with higher baseline ADA titers (>1:1000) consistently demonstrating more prolonged systemic exposure compared with those having lower ADA titers, particularly evident at the higher dose levels (0.3 and 0.6 mg/kg) (**Supplementary Table 3**), where the effect on the elimination phase was most pronounced, indicating a potential dose-dependent effect. VTX-PID demonstrated dose-dependent increases in plasma exposure (C_max_ and AUC) across the four ascending-dose cohorts (**Table 3**). C_max_ showed dose proportionality, as evidenced by consistent dose-normalized C_max_ values across all cohorts. Systemic exposure (AUC from time zero to the last quantifiable concentration [AUC_last_]) exhibited a trend toward greater than dose-proportional exposure at higher doses. Limited data to calculate AUC from time zero extrapolated to infinity (AUC_inf_) were available because the profiles failed to meet acceptability criteria, particularly for Cohorts 1 and 4, indicating challenges in fully characterizing the elimination phase at these dose levels. Terminal elimination half-life (t_½_) appeared dose dependent, increasing from 4.84 hours at the lowest dose to 41.01 hours at the highest dose (**Table 3**). Moderate to high variability was observed in most PK parameters, particularly for t_½_ (geometric coefficient of variation [CV]% up to 874.5%), indicating substantial interparticipant differences in drug disposition.

**Table 3:** Summary statistics of VTX-PID plasma pharmacokinetic parameters across all four cohorts (PK analysis set).

PK parameter	Statistic	Cohort 1 VTX-PID (0.075 mg/kg) (*n* = 6)	Cohort 2 VTX-PID (0.15 mg/kg) (*n* = 6)	Cohort 3 VTX-PID (0.3 mg/kg) (*n* = 9)*	Cohort 4 VTX-PID (0.6 mg/kg) (*n* = 6)
C_max_, μg/mL	Arithmetic mean (SD)	1.83 (0.24)	3.64 (0.44)	7.22 (1.19)	13.68 (1.63)
t_max_, h	Median (range)	0.75 (0.74–1.51)	0.78 (0.51–1.49)	0.75 (0.50–1.01)	1.13 (1.00–1.35)
DNC_max_, kg*μg/mL/mg	Arithmetic mean (SD)	22.81 (3.05)	24.24 (2.95)	23.48 (3.94)	22.59 (2.46)
AUC_last_, h*μg/mL	Arithmetic mean (SD)	11.74 (15.59)	46.65 (63.16)	92.45 (88.27)	164.3 (82.41)
AUC_inf_, h*μg/mL	Arithmetic mean (SD)	NC (NC)	19.62 (2.27)^†^	103.3 (106.0)^‡^	NC (NC)^§^
t_½_, h	Arithmetic mean (SD)	10.43 (17.73)	18.99 (31.10)^‖^	28.86 (28.60)^¶^	88.13 (59.06)^‖^
	Geometric mean (geometric CV%)	4.84 (163.5)	7.16 (282.1)	12.55 (326.9)	41.01 (874.5)
CL, mL/h/kg	Arithmetic mean (SD)	NC (NC)	7.72 (0.96)^†^	6.88 (4.97)^‡^	NC (NC)^§^
V_z_, mL/kg	Arithmetic mean (SD)	NC (NC)	38.03 (3.91)^†^	77.93 (47.78)^‡^	NC (NC)^§^

*Three nonrandomized participants were included and treated with 0.3 mg/kg VTX-PID (Cohort 3) after the end of the escalation phase (Day 14 of the last participant for Cohort 4) along with randomized participants. ^†^*n* = 3. ^‡^*n* = 7. ^§^*n=1.* ^‖^*n=4.* ^¶^*n=8*. AUC_inf_, AUC from time zero extrapolated to infinity; AUC_last_, AUC from time zero to the last quantifiable concentration; C_max_, maximum observed concentration; CL, apparent clearance following intravascular administration; CV, coefficient of variation; DNC_max_, dose−normalized C_max_; NC, not calculable; SD, standard deviation; t_½_, apparent terminal elimination half-life; t_max_, time corresponding to occurrence of C_max_; V_z_, apparent volume of distribution during terminal phase following intravascular dosing.

In the statistical analysis to assess dose proportionality (power model), C_max_ estimated slope (B1) was 0.9715 (95% confidence interval [CI] 0.8986–1.0444), demonstrating a strong correlation between log_e_ (dose) and log_e_ (C_max_) as evidenced by the high R-square value of 0.968 (**Table 4**). C_max_ increased proportionally with increasing dose within the studied dose range. AUC_inf_ analysis only included 11 of 27 possible observations. The dose proportionality estimated slope (B1) for AUC_inf_ was 1.0059 with a corresponding 95% CI of –0.5763 to 2.5882 and a very low R-square value (0.187), indicating higher variability in the AUC_inf_ data (**Table 4**).

**Table 4:** Statistical analysis to assess dose proportionality (Power model)* of plasma pharmacokinetic parameters of VTX-PID (PK analysis set).

Parameter	n	B0	95% CI for B0	B1 (SE)	95% CI for B1	R^2^ value
C_max_, μg/mL	27	3.1212	2.9986–3.2439	0.9715 (0.0354)	0.8986–1.0444	0.968
AUC_inf_, h*μg/mL	11	5.1541	2.9572–7.3509	1.0059 (0.6995)	–0.5763–2.5883	0.187

*Dose proportionality is assessed using the power model. In this power model analysis, it was assumed that the natural logarithm of the PK variable was linearly related to the natural logarithm of dose as in the following equation: log (PK parameter) = B0 + B1 * log (dose). This model was fit with a simple linear regression to produce an estimate and a 95% CI (two-sided) for the slope coefficient B1. Dose proportionality based on the power model is accepted (“not rejected, “ in terms of statistical inference) if the 95% CI (two-sided) of the slope for each of the PK parameters includes 1. For dose independence, the slope of the regression line is equal to 0. AUC_inf_, area under the concentration-time curve from time zero extrapolated to infinity; B0, intercept; B1, slope; CI, confidence interval; C_max_, maximum observed concentration; n, number of observations; PK, pharmacokinetics; SE, standard error.

### 3.4 Pharmacodynamics

The PD analysis set included 27 participants who received VTX−PID across the four cohorts and eight who received placebo.

#### 3.4.1 Total IgG

**Figure 2:**
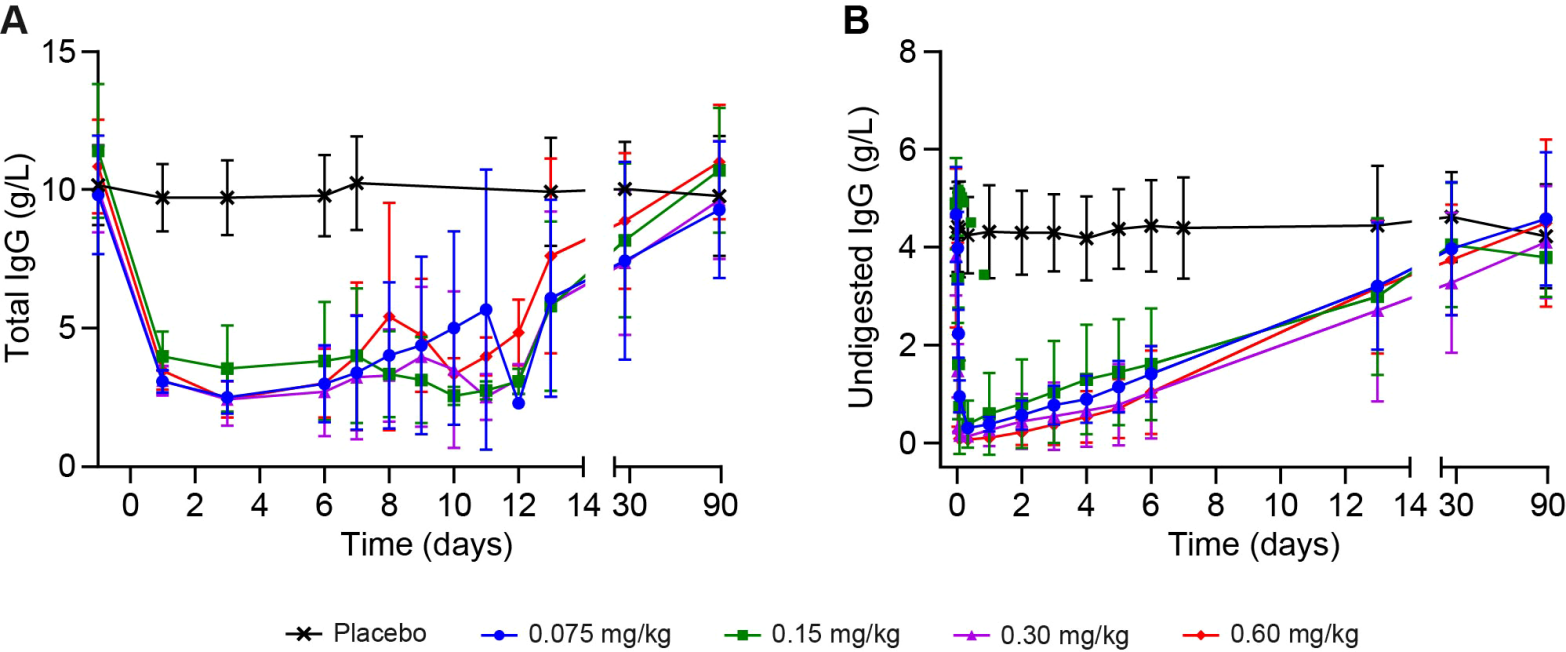
Pharmacodynamic analysis of the effect of VTX-PID on circulating concentrations of **(A)** total IgG and **(B)** undigested IgG over 90 days post dose across all four cohorts. Means ± standard deviation are represented. IgG, immunoglobulin G.

All VTX-PID dose cohorts demonstrated early response with substantial and rapid reductions in total IgG concentrations by 1 day post dose, with mean decreases from baseline ranging from –6.723 g/L to –7.415 g/L, compared with the placebo group, which showed minimal change. Maximum IgG reduction was observed around 3 days post dose, with mean total IgG reductions from baseline ranging from –7.302 g/L to –8.392 g/L (**Figure 2A**). By 13 days post dose, partial recovery of total IgG levels was observed across all VTX−PID treatment cohorts. None of the participants required the IV Ig administration, as they all demonstrated a clear trend toward IgG recovery prior to 13 days post dose. Further increase was noted by 27 days post dose across dose cohorts. By the end-of-study visit, all VTX−PID treatment cohorts returned to near-baseline IgG levels, with changes from baseline (CFB) from –0.703 to +0.167 g/L.

#### 3.4.2 Total undigested IgG

VTX-PID demonstrated dose- and time-dependent reduction of total undigested IgG with rapid onset (within 30 minutes) and sustained effect through approximately 6 days post administration across all cohorts (**Figure 2B**). At 30 minutes, the mean CFB ranged from –0.683 g/L in Cohort 1 to –2.332 g/L in Cohort 3. In contrast, the pooled placebo group showed minimal change (mean CFB, 0.108 g/L). Maximum reductions in total undigested IgG were observed at 8 hours post dose, with the timing of maximum effect appearing to be inversely related to dose. Mean CFB at 8 hours post dose ranged from –3.671 g/L to –4.507 g/L across the VTX−PID cohorts. The 0.3 mg/kg and 0.6 mg/kg doses demonstrated the most pronounced early reductions, with mean concentrations decreasing to near the LLOQ at 1 hour post dose of 0.339 g/L and 0.232 g/L, respectively. Substantial reductions in total undigested IgG (defined as >3 g/L below baseline) were noted through approximately 6 days post dose across all VTX-PID cohorts. All cohorts showed gradual recovery immediately after the maximal reduction. By 13 days post dose, mean reductions were much less pronounced (range from –0.803 g/L to –1.565 g/L for the four dose cohorts); by 27 days post dose they were minimal (ranging from −0.243 to −0.840 g/L), with levels largely returning to baseline by the end of the study. A clear dose−response relationship was observed for the onset of action, with higher doses achieving more rapid reductions in total undigested IgG levels and longer duration of low total undigested IgG levels.

#### 3.4.3 F(ab')_2_ fragment

**Figure 3:**
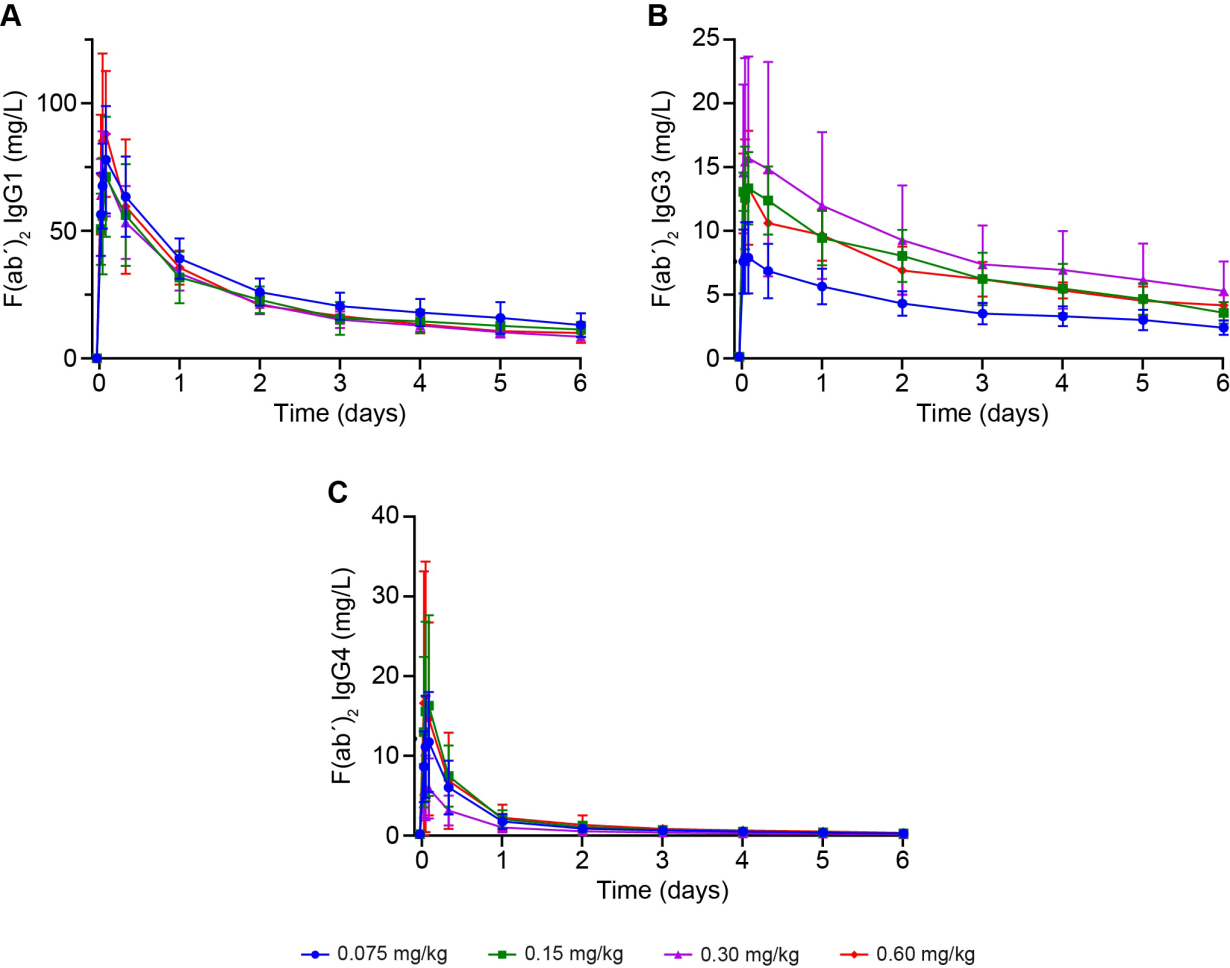
Pharmacodynamic analysis of the effect of VTX-PID on F(ab')_2_ fragments up to 6 days post dose across all four cohorts for **(A)** IgG1, **(B)** IgG3, and **(C)** IgG4. Means ± SD are represented. When a result below the limit of quantification was obtained, a value of LLOQ value/2, i.e. 0.125 µg/mL, was used for representation. IgG, immunoglobulin G; LLOQ, lower limit of quantification.

Following VTX-PID administration, all cohorts demonstrated rapid increases in F(ab')_2_ fragment concentrations across all three signature peptides (IgG1, IgG3, and IgG4), with elevations observed by the first assessment at 30 minutes post dose (**Figure 3**). Peak F(ab')_2_ fragment concentrations generally occurred at 2 hours post dose across all cohorts.

For IgG3, there was a dose-dependent increase in F(ab')_2_ fragments observed in the 0.075, 0.15, and 0.3 mg/kg cohorts, with no additional benefit at the 0.6 mg/kg dose, indicating a plateau effect at doses ≥0.3 mg/kg. Following peak, concentrations declined gradually 24 hours post dose and 48 hours post dose (**Figure 3B**); IgG3 demonstrated the most consistent dose-response relationship.

IgG1 demonstrated substantially higher concentrations and maintained the most sustained elevation compared with the other peptides. Mean concentrations remained elevated at 24 and 48 hours post dose (**Figure 3A**).

IgG4 declined most rapidly, with low concentrations by 24 hours post dose and very low levels by 48 hours post dose (**Figure 3C**). IgG4 showed the most variable response and the shortest duration of effect.

All peptides were undetectable in the pooled placebo group throughout the study.

#### 3.4.4 Anti-AAV3B TAb levels over time

**Figure 4:**
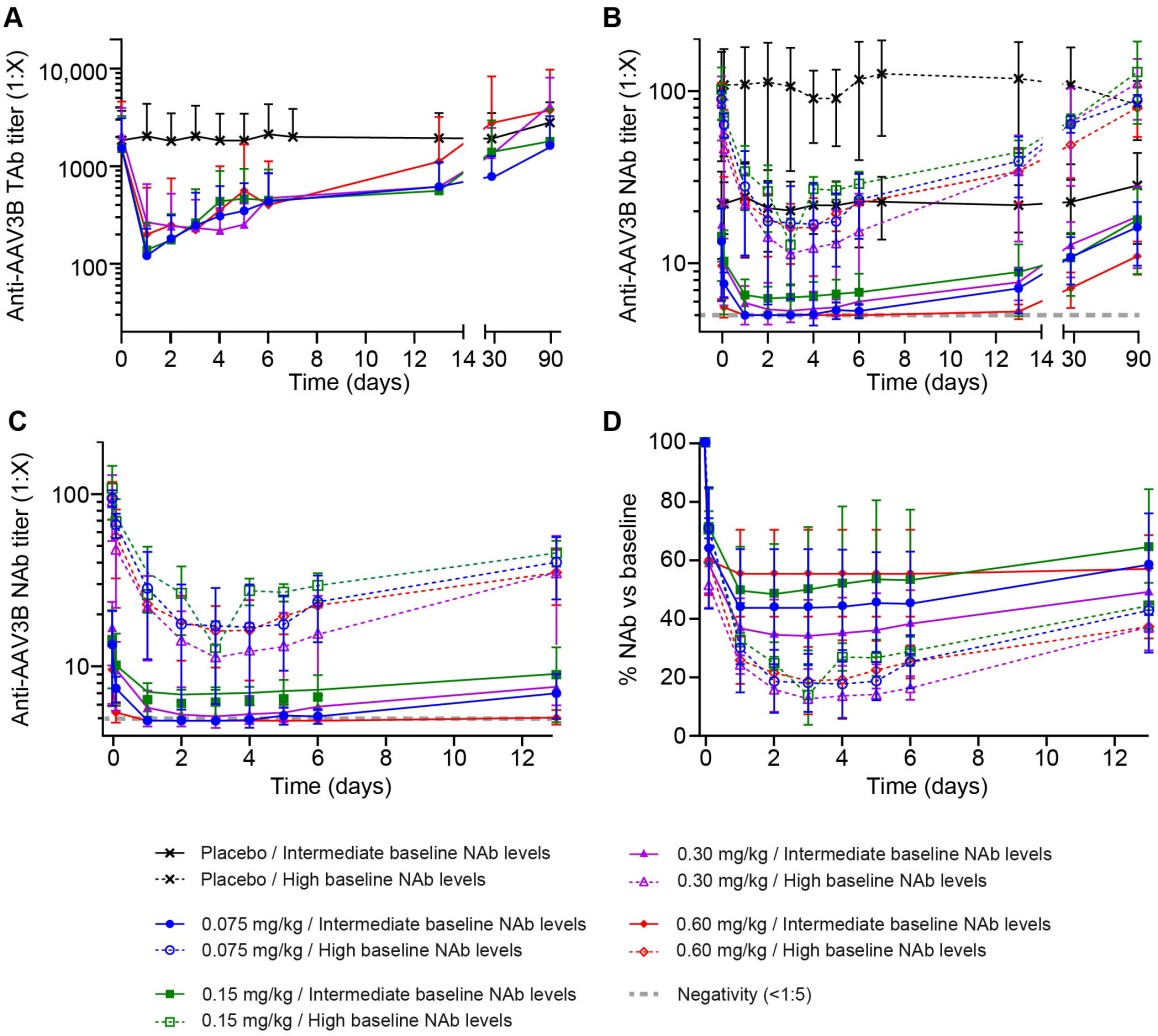
Pharmacodynamic analysis of the effect of VTX-PID on anti-AAV3B TAb and anti-AAV3B NAb levels over time. **(A)** TAbs up to Day 90 post dose, **(B)** NAbs up to Day 90 post dose, **(C)** focus on NAbs up to 13 days post dose, **(D)** results of NAbs in % (normalized with NAbs baseline) up to 13 days post dose. The dashed gray line represents the negativity threshold at <1:5. For NAbs only: solid and dashed lines and closed and open symbols represent the subpopulations with intermediate or high baseline NAb levels respectively. Means ± standard deviation are represented. AAV3B, adeno-associated virus serotype 3B; NAb, neutralizing antibody; TAb, total antibody.

Following VTX-PID administration, all VTX-PID cohorts demonstrated a rapid and substantial reduction in anti-AAV3B TAb levels by 1 day post dose, with mean titers decreasing from baseline to 1:120.7 (range, 1:33–1:295) in Cohort 1, 1:139.0 (range, 1:20–1:284) in Cohort 2, 1:265.3 (range, 1:20–1:1034) in Cohort 3, and 1:199.5 (range, 1:316–1:7194) in Cohort 4 (**Figure 4A**). In contrast, participants in the pooled placebo group maintained consistently high TAb levels throughout the study period. From 1 to 5 days post dose, TAb levels remained relatively stable, but some participants across all VTX−PID dose cohorts showed slight increases. During the recovery phase (6 days post dose to end of study), TAb levels gradually increased, with the extent of recovery varying by dose cohort. During the recovery phase, lower dose cohorts (1 and 2) showed a more gradual return toward baseline levels, with end-of-study mean values only modestly exceeding baseline measurements, while in Cohorts 3 and 4, mean TAb levels substantially exceeded baseline values (**Figure 4A**).

#### 3.4.5 Anti-AAV3B NAb levels over time

VTX-PID administration resulted in rapid dose-dependent reductions in anti-AAV3B NAb titers across all cohorts, with effects most pronounced in the higher dose cohorts (**Figures 4B, C**). NAb titers in the placebo group remained stable throughout the study. At 24 hours post dose, mean NAb titers had decreased from baseline to 1:16.50 titer (range, 1:5.00–1:47.34) in Cohort 1, 1:15.77 titer (range, 1:5.00–1:43.99) in Cohort 2, 1:11.24 titer (range, 1:5.00–1:33.85) in Cohort 3, and 1:10.88 titer (range, 1:5.00–1:23.77) in Cohort 4. NAb levels continued to decline through 2–3 days post dose, with many participants across treatment cohorts approaching the negativity threshold of <1:5 (**Figures 4B, C**). This suppression persisted through approximately 6 days post dose, after which NAb levels gradually rebounded, approaching near-baseline values by the end of the study (**Figure 4B**). A dose−dependent trend was observed in the magnitude and duration of NAb suppression, with the 0.6 mg/kg cohort showing the most pronounced and sustained reduction, with the mean titer reduced from baseline 1:36.47 to 1:10.88–1 day post dose. The 0.3 mg/kg cohort exhibited similar but less pronounced effects. The 0.075 and 0.15 mg/kg cohorts showed shorter-lasting NAb suppression, with a quicker return to baseline levels.

Baseline stratification by intermediate or high NAb levels significantly influenced the PD response (**Figure 4D**). Participants with high NAb levels showed greater reductions but maintained higher absolute NAb levels throughout the treatment period compared with those with intermediate NAb levels. In Cohort 1, participants with high NAb levels demonstrated a substantial mean titer reduction from baseline (1:89.82) to 2 days post dose (1:17.57), representing an approximately 5.1-fold reduction. In contrast, participants with intermediate NAb levels in the same cohort saw a more modest mean reduction from baseline (1:13.42) to 2 days post dose (1:5.00), a decrease of approximately 2.7-fold. This pattern was consistent across all dose cohorts.

Participants with high NAb levels also demonstrated a more pronounced rebound effect to near mean baseline levels at the end of the study. This pattern was consistent across all dose cohorts, with this differential recovery pattern even more pronounced in Cohort 3. Some participants with intermediate NAb levels across all cohorts reached the minimal detectable levels (<1:5 titer), determined as the noninhibitory level, by 2 to 3 days post dose (some by 1 day post dose), showed more gradual recovery of NAb levels, and maintained lower absolute NAb levels throughout the study. Conversely, participants with high NAb levels maintained detectable NAbs above the negativity threshold throughout the study.

**Figure 5:**
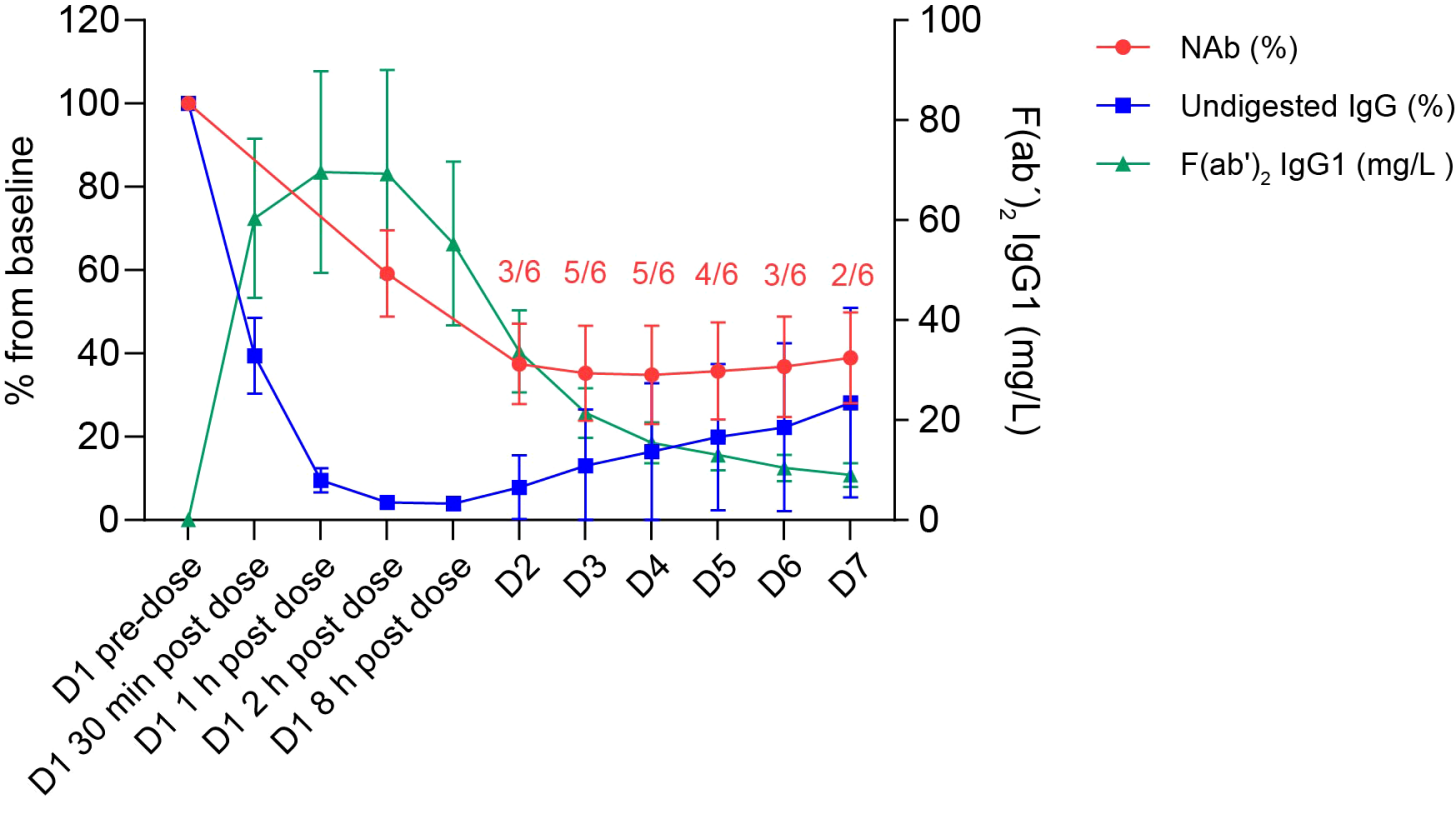
Summary graph for the selected dose 0.3 mg/kg (Cohort 3) and the intermediate NAb level subpopulation. For NAbs, the number of participants reaching a titer ≤1:5 (negativity) out of the six treated participants in the 0.3 mg/kg cohort is indicated above in red from Day 2 to Day 7. Means ± standard deviation are represented. D, day; NAb, neutralizing antibody; TAb, total antibody.

The proportion of participants achieving negative NAb status differed among dose cohorts, with the highest rate in Cohort 4 (4; 66.7%), followed by Cohort 3 (5; 55.6% by 2 days post dose), Cohort 1 (3; 50.0%), and Cohort 2 (1; 16.7%). No participants with high baseline NAb levels in any cohort achieved this conversion. By 1 day, postdose conversion rates among those with intermediate NAb levels were 100% in Cohort 1, 25% in Cohort 2, 50% in Cohort 3 (83.3% by 2 days post dose), and 100% in Cohort 4. Among 13 participants who seroconverted to negative NAb status (<1:5), the majority (11 [84.6%]) did so 1 day post administration, with the remaining conversions occurring between 2 and 3 days post dose (one participant each, both in the 0.3 mg/kg cohort). The pooled placebo group did not achieve negative anti−AAV3B NAb status. VTX-PID demonstrated a dose-dependent effect on the median duration of negative anti-AAV3B NAb status in participants who achieved this conversion, with the longest duration of 13 days observed at the highest dose level (0.6 mg/kg, Cohort 4) and a median duration of 10 days, while in Cohort 1, the median duration was 1 day compared with 0 days for Cohort 2. The longest duration of negative anti−AAV3B NAb status in Cohort 3 was 12 days. Response by baseline NAb status varied across cohorts with intermediate levels; in Cohort 3, the median duration was 1.5 days (range, 0–12), with four participants who converted to negative NAb status. Duration of negative anti-AAV3B NAb status across cohorts is shown in **Supplementary Table 4**. The 0.3 mg/kg dose provided optimal PD effects, particularly in participants with intermediate baseline anti-AAV3B NAbs (**Figure 5**).

### 3.5 Immunogenicity

**Figure 6:**
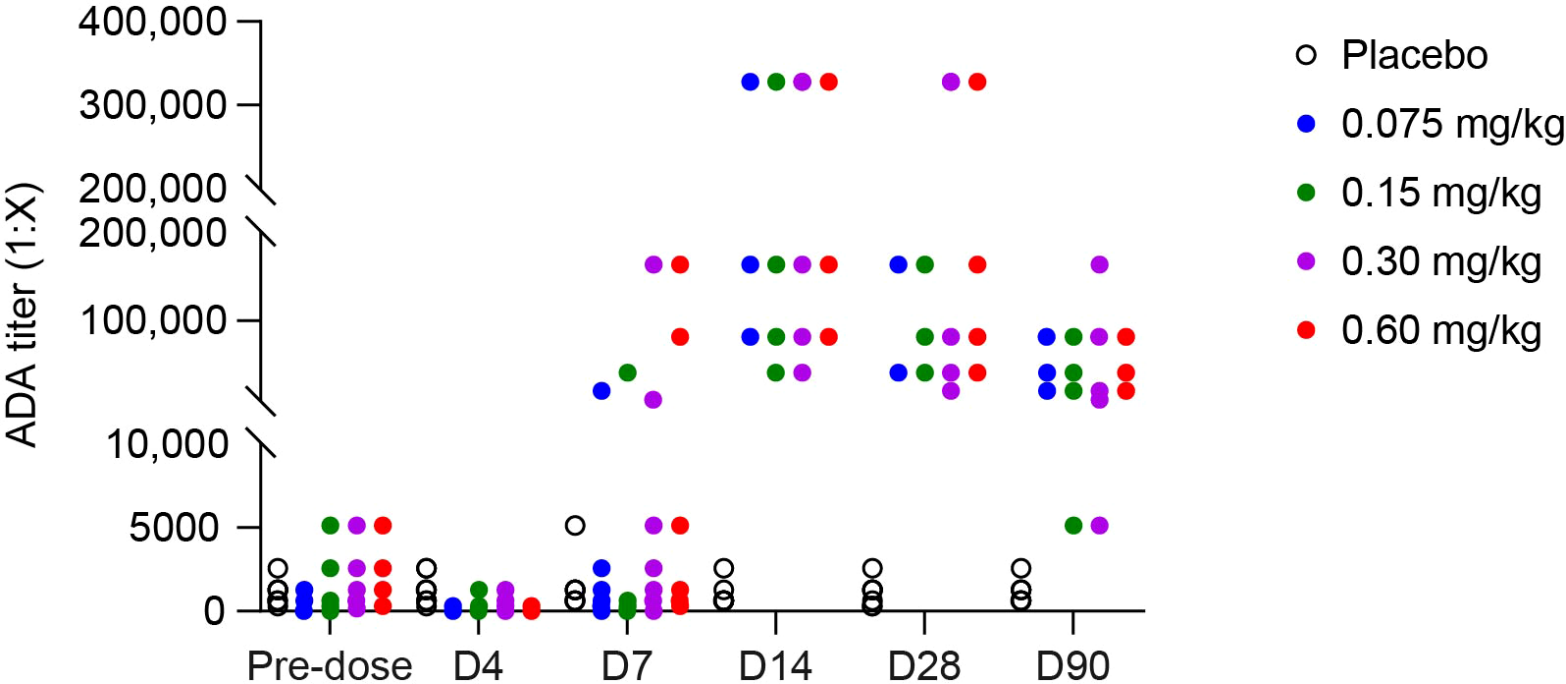
Comparison of antidrug antibody (ADA) titers before and after administration of different doses of VTX-PID across all cohorts. Individual data are represented. When result was negative (no titer value), an arbitrary value of 1 was used for representation. D, day.

At baseline, 30 of 35 participants (85.7%) had pre−existing ADA against VTX−PID, 18 of them with ADA titers >1:1000. By Day 4 (3 days post dose), all participants showed a marked reduction in ADA titers versus baseline levels, resulting in a shift in ADA titer distribution, with the majority of participants having no more detectable ADAs across all VTX−PID cohorts (**Figure 6**). By Day 7 (6 days post dose), ADA positivity rates increased in VTX−PID cohorts; ADA titers >1:1000 were observed in three participants (50.0%) in Cohort 1, one (16.7%) in Cohort 2, six (66.7%) in Cohort 3, and four (66.7%) in Cohort 4. A significant ADA titer increase in postdose samples compared with baseline was indicative of a VTX−PID treatment−boosted ADA response. ADA positivity peaked by Day 14 (13 days post dose) in all VTX−PID cohorts (six participants [100%] in all cohorts except Cohort 3, in which eight of nine [88.9%] were positive), while the placebo group remained unchanged according to criterion for treatment-boosted immune reaction. This immunogenicity profile persisted through Day 28 (27 days post dose), with high ADA positivity rates maintained across all VTX−PID cohorts. By the end−of−study visit (Day 90), the pattern remained consistent, with persistent ADA positivity in VTX−PID cohorts and no change from baseline in the placebo group.

## 4 Discussion

Preexisting anti-AAV NAbs pose a major challenge to successful AAV-based gene delivery due to transduction impediment and, despite various strategies to overcome NAbs, seropositive individuals remain largely ineligible for systemic AAV-based therapies (4, 8, 12, 16, 23). VTX-PID, a recombinant human IdeS, demonstrated treatment opportunity by reducing anti-AAV NAbs titers (17). In this first-in-human NAVIgATE study, VTX-PID was evaluated at single ascending doses in healthy male participants for safety, PK, and PD. VTX-PID demonstrated a favorable benefit-risk safety profile along with no serious AEs or discontinuations. AEs were manageable with or without concomitant medications, were generally transient, and resolved without sequelae. Across the tested dose range of 0.075–0.6 mg/kg (Cohorts 1–4), VTX-PID showed rapid initial distribution after administration, reaching peak concentrations within a median time to C_max_ of 0.75–1.13 hours, followed by a multiphasic decline. VTX-PID PK was impacted by pre-existing ADAs, with high baseline titers (>1:1000) linked to reduced clearance, resulting in increased exposure (AUC) and prolonged elimination half-life, especially at higher doses, although no PD effect was observed in IgG or NAb reduction. Increased ADA levels at baseline can contribute to nonlinear PK by enhancing VTX-PID clearance (clearing ADAs) or through neutralizing ADAs binding to active site of the enzyme, which could reduce catalytic activity, leading to loss of effect—and increase AUC by blocking target-mediated drug disposition–mediated elimination. Further analyses of the impact of ADA on VTX-PID PK are needed. Considering that, we observed a decrease in ADA levels leading to negativity in most subjects at Day 3 following VTX-PID injection to subjects, re-administration of VTX-PID could be considered 1 or up to 3 days following the first administration to increase further the reduction of anti-AAV NAb levels. This difference regarding PK elimination is also linked to the use of a fully validated and sensitive assay (LLOQ <0.5 µg/mL) measuring bound and unbound VTX-PID. This assay enables the detection of lower VTX-PID concentrations with greater specificity than previously published (24), thereby providing improved understanding of the elimination profile. VTX-PID led to rapid and substantial reductions in total IgG, undigested IgG, and anti-AAV3B TAb and NAb levels, with corresponding increases in F(ab')_2_ fragments across all cohorts. Dose-dependent reductions were observed for anti-AAV3B NAbs and total undigested IgG. Baseline anti-AAV3B NAb status significantly influenced NAb response to treatment, with substantially better responses observed across all dose cohorts in participants with intermediate baseline anti-AAV3B NAb levels (<1:45) compared with those with high baseline anti-AAV3B NAb levels (≥1:45), among whom the response did not achieve levels below the threshold for non-inhibitory activity (≤1:5).

The primary endpoint of this study was the safety profile of VTX-PID across multiple dose cohorts (0.075–0.6 mg/kg). All protocol-defined dose levels were successfully escalated, and every participant received the intended dose. The study was suspended temporarily when one participant in Cohort 4 met predefined stopping criteria of ALT or AST >3 × ULN accompanied by ALP >1.5 × ULN; the study resumed after a protocol amendment, which introduced a prophylactic mitigation regimen with concomitant prednisolone administered during the first 2 days. VTX-PID demonstrated a manageable safety profile across all four cohorts, with mostly mild to moderate TEAEs, and only two participants experienced severe events. TEAEs considered related to VTX-PID occurred in 57.1% of participants, with musculoskeletal disorders (particularly myalgia [34.3%] and arthralgia [25.7%]) being most common. This safety profile aligns with previous clinical experience with bacterial IdeS enzymes (24). Infusion-related reactions, a known effect of IdeS, occurred in 11.4% of participants, primarily in the highest dose cohort without-corticosteroid premedication (three of four cases) (22, 24, 27). Notably, among the six participants premedicated with corticosteroids (three in Cohort 4 and three nonrandomized in Cohort 3), only one in Cohort 3 experienced an infusion-related reaction, suggesting that prophylactic corticosteroid administration may provide a protective effect against these reactions. Transient elevations in liver enzymes, specifically ALT, and transient proteinuria were observed, particularly in higher dose cohorts, with all abnormalities resolving spontaneously within 3–4 days and fully normal by end of study without specific intervention. Despite the implementation of corticosteroid prophylaxis (60 mg prior to VTX-PID dosing and 30 mg 24 hours post dose), one participant experienced a significant increase in ALT. This indicates that, although this corticosteroid regimen was effective in managing infusion-related reactions, it was not effective to prevent the biochemical hepatic alteration (ALT increase & CRP increase) occurring several days after infusion in the setting of a systemic inflammatory response syndrome which peaked around 5–7 days post dose. In AAV gene therapy protocols, prolonged corticosteroid treatment regimens are generally employed to manage AAV-related reactions (28); a similar treatment approach would likely also be beneficial to VTX-PID pretreatment, however, it was not ethically and medically justifiable to administer in healthy volunteers. Transient proteinuria was also reported in the clinical evaluations of IdeS, while ALT elevation was observed with KJ103, an IdeE variant with IgG-cleaving activity (6, 24). Proteinuria very likely reflects the clearance of IgG cleavage products from circulation after cleavage by VTX-PID/IdeS (24). Laboratory evaluations revealed a treatment-related, dose-dependent transient rise in the inflammatory marker high-sensitivity C-reactive protein, with elevations most pronounced at higher VTX-PID doses; all inflammatory responses resolved by the end of the study, without intervention. Safety findings, notably transient liver enzyme elevations at 0.6 mg/kg (Cohort 4), indicated that this highest dose may confer a greater likelihood of ALT elevations, particularly when accompanied by systemic high-sensitivity C-reactive protein elevation which was deemed unexpected, not predictable, likely idiosyncratic, well tolerated and self−resolving, resulting in the decision to use the 0.3 mg/kg dose for the additional 3 non−randomized subjects. VTX-PID PK parameters of C_max_ demonstrated dose proportionality across the four ascending dose cohorts consistent with high bioavailability after intravenous administration; overall plasma exposure (AUC) also rose with dose. Quantifiable concentrations were maintained through the 144−hour sampling period in one participant receiving 0.3 mg/kg and 0.6 mg/kg, whereas lower dose cohorts reached or fell below the LLOQ earlier. Dose proportionality based on the power model was achieved as the 95% CIs (two-sided) of the slope, for each of the PK parameters (C_max_ and AUC_inf_), include 1; however, due to high interparticipant variability and the limited number of calculable parameters, definitive conclusions regarding dose proportionality for AUC_inf_ cannot be drawn. Pre-existing ADA titers (≤1:1000 or >1:1000) appear to influence VTX-PID PK, similar to IdeS (24); higher baseline ADA titers (>1:1000) were consistently associated with increased systemic exposure parameters (C_max_ and AUC_last_) and prolonged elimination (t_½_) compared with those with lower titers, particularly at higher dose levels, indicating a potential dose-dependent effect. Overall, VTX−PID exhibited a PK profile, using a more sensitive and validated assay, broadly consistent with that of IdeS, validating the predictive utility of preclinical models and supporting 0.3 mg/kg as the recommended dose for future clinical studies (22, 24, 29).

VTX−PID induced rapid, dose−dependent reductions in total and undigested IgG, with maximal reduction around 3 days post dose, and demonstrated a clear trend of IgG recovery, consistent with the phase I Idefirix^®^ study, other IdeS studies, and preclinical data on IdeS (17, 22, 24, 29). Across cohorts, F(ab')_2_ fragment levels generally peaked at 2 hours post dose, with 0.3 mg/kg yielding the most favorable PD profile for IgG3 and IgG1. It should be noted that only fragments from IgG1, IgG3, and IgG4 were measured, representing about 68% of the total IgG population (30). VTX−PID produced rapid, robust decreases in anti-AAV3B TAb levels across all dose cohorts, with higher doses driving greater and longer responses. The VTX−PID PD response with respect to reduction in anti−AAV3B NAbs levels was rapid, with NAb levels decreasing to negative anti-AAV3B NAb status (titer <1:5) observed as early as 30 minutes post dose in some participants and peak effect generally occurring within 1–3 days post dose. The magnitude and duration of these effects exhibited dose dependency, with higher doses generally providing more pronounced and sustained responses. The achievement of negative NAb status occurred primarily 1 day post dose, indicating prompt PD activity of VTX−PID on NAb levels following administration. The lack of conversions beyond 3 days post dose suggests that participants who did not respond were unlikely to improve within the 7−day assessment period. Participants with high baseline NAbs (titer ≥1:45) did not achieve reduction of anti-AAV3B NAbs to noninhibitory levels (titer ≤1:5) in any of the cohorts, thus failing to create an effective treatment window for AAV dosing. Achieving ≤1:5 titer is crucial, as this threshold was determined by the sponsor as noninhibitory for efficient AAV3B vector transduction, allowing otherwise ineligible patients (with pre-existing NAbs levels up to ≤1:45) to become eligible for AAV therapy. Parallel PD responses between anti-AAV3B TAb and NAb levels after VTX-PID administration suggest TAbs could be a practical surrogate for NAb status, which, given the faster TAb assay turnaround, could streamline decisions on optimal AAV gene therapy administration following VTX-PID.

These findings suggest that both dose selection and baseline NAb status are critical factors in maximizing the PD effect of VTX-PID on reduction of anti-AAV3B NAbs. This means that for every AAV serotype and corresponding NAb assay, we need to understand how NAb-positive titers are distributed in patients so that we can estimate how many might become eligible for AAV gene therapy after VTX-PID treatment. Notably, during the screening process, participants exhibiting high NAb levels were observed less frequently than those with intermediate NAb levels; indeed, among the 580 screened subjects, 391 (i.e 67%) were negative, 110 (19%) had an intermediate NAb titer, and 79 (14%) had a high NAb titer. Consequently, the lack of responsiveness to VTX-PID observed in participants with high NAb titers may apply to only a small proportion of the overall patient population.

Based on the overall benefit-risk assessment, the 0.3 mg/kg dose of VTX-PID (Cohort 3) was identified as the recommended dose for future clinical studies. This dose provided the optimal PD profile in this population while maintaining a manageable safety profile, as determined by the number of participants reaching negative anti−AAV3B NAb levels (titers ≤1:5), the duration of this negativity, the increases in F(ab')_2_ fragment concentrations, and the reduction of total undigested IgG observed in participants who had intermediate baseline anti-AAV3B NAb levels. In this subpopulation, the rapid decrease in IgG levels due to their digestion by VTX-PID resulted in concomitant production of F(ab')_2_, which was almost cleared by 6 days post dose. Anti-AAV3B NAbs, which are mainly IgG, were thus cleaved by VTX-PID, leading to NAbs below or at the non-inhibitory threshold (≤1:5) for several days in the majority of participants; this constitutes a window of opportunity for an optimal systemic treatment with an AAV3B-based gene therapy. In this cohort, the duration of this window extended up to 5 days, with most participants experiencing a 2- to 5−day treatment window. The reduction of NAb levels at 0.3 mg/kg VTX-PID potentially broadens the treatable population and establishes a predictable window of 2–5 days for AAV3B administration in comparison with lower doses of VTX-PID. This could potentially enable treatment in approximately 52%–89% of individuals across diverse populations for whom pre−existing anti−AAV3 NAbs pose a major barrier to effective AAV vector–mediated gene therapy (31, 32). Additionally, cross−reactivity between AAV serotypes occurs frequently, although it is not uniformly observed across all serotypes (7, 13, 31). This underscores the need for validated NAb assays to evaluate serum responses to each AAV serotype at baseline and after any reduction strategy to determine therapeutic benefit and define the eligible patient population. These results support the potential utility of VTX-PID as a pretreatment strategy to safely and temporarily reduce circulating anti-AAV3B antibody levels, potentially allowing treatment of previously ineligible patients with AAV-based gene therapy. We demonstrated *in vitro* the ability of VTX-PID to effectively cleave IgG directed against various AAV serotypes. Indeed, human sera (n=4) with known neutralizing antibody (NAb) titers against these serotypes were digested with VTX-PID, and the residual neutralizing activity was assessed. The results showed a comparable reduction in neutralizing activity following incubation with VTX-PID for AAV-Anc80, AAV8, AAV2, AAV3B, and AAV9 (17). Such a pretreatment strategy allowed gene therapy treatment for one patient with the Crigler-Najjar syndrome with AAV8 (33).

These first data from the NAVIgATE study in healthy male participants further support the use of VTX-PID as a pretreatment to transiently deplete anti-AAV3B NAbs in naturally immunized participants, which represents a promising approach to broaden patient eligibility for AAV-based gene therapies. To extend the use of VTX-PID to gene therapies using AAV serotypes other than AAV3B, it may be necessary to confirm the window of opportunity using specific validated NAb assays; the NAVIGATE serum bank has been created for this purpose. Future studies could include PK/PD modeling to improve responder predictivity. Other studies could include validation assays for each AAV vector to ensure proper threshold definition and dose response assessment using baseline values for each patient and predictive assays involving both TAb and NAb measurements.

Consequently, initiating future gene therapy clinical studies with subjects exhibiting the lowest baseline NAb levels, and structuring enrollment to stratify by NAb titer, will maximize success rates and enable refinement of the predictive model with clinically relevant outcome data. Collectively, these data reinforce the potential applicability of VTX-PID as an enabling platform for gene therapy trials, particularly in settings where pre-existing AAV immunity would otherwise preclude treatment.

## Data Availability

The original contributions presented in the study are included in the article/**Supplementary Material**. Further inquiries can be directed to the corresponding author.
