# Correction: VTX-PID as a novel recombinant immunoglobulin G–degrading enzyme (IdeS) for efficient AAV-based gene therapy in participants with neutralizing antibodies: results of the phase I first-in-human NAVIgATE study

**DOI:** 10.3389/fimmu.2026.1899342

**Published:** 2026-06-17

**Authors:** Bernard Benichou, Rainard Fuhr, Elodie Vernadal, Sonia Valero, Blanche Tamarit, Veronica Ferrer, Gloria Gonzalez-Aseguinolaza, Anne Douar, Marc Froissart, Annelise Brossel, Simone Floettmann, Francesca Del Bene, Céline Bouquet, Jean-Philippe Combal

**Affiliations:** 1 Vivet Therapeutics SAS, Paris, France; 2 Parexel International GmbH, Berlin, Germany; 3 DNA & RNA Medicine Division, Gene Therapy for Rare Diseases Department, Center for Applied Medical Research (CIMA), University of Navarra, IdisNA, Pamplona, Navarra, Spain; 4 Vivet Therapeutics SL, Pamplona, Spain; 5 Lausanne University Hospital and University of Lausanne, Lausanne, Switzerland; 6 Parexel International SRL, Milan, Italy

**Keywords:** adeno-associated virus, gene therapy, IgG, Imlifidase, neutralizing antibodies, total antibodies, VTX-PID

There was a mistake in **Figure 4** as published. The **Figure 4C** has been updated to show the correct threshold of negativity, and the mention of negativity threshold has been added in the legend. The corrected **Figure 4** appears below.

**Figure 4:**
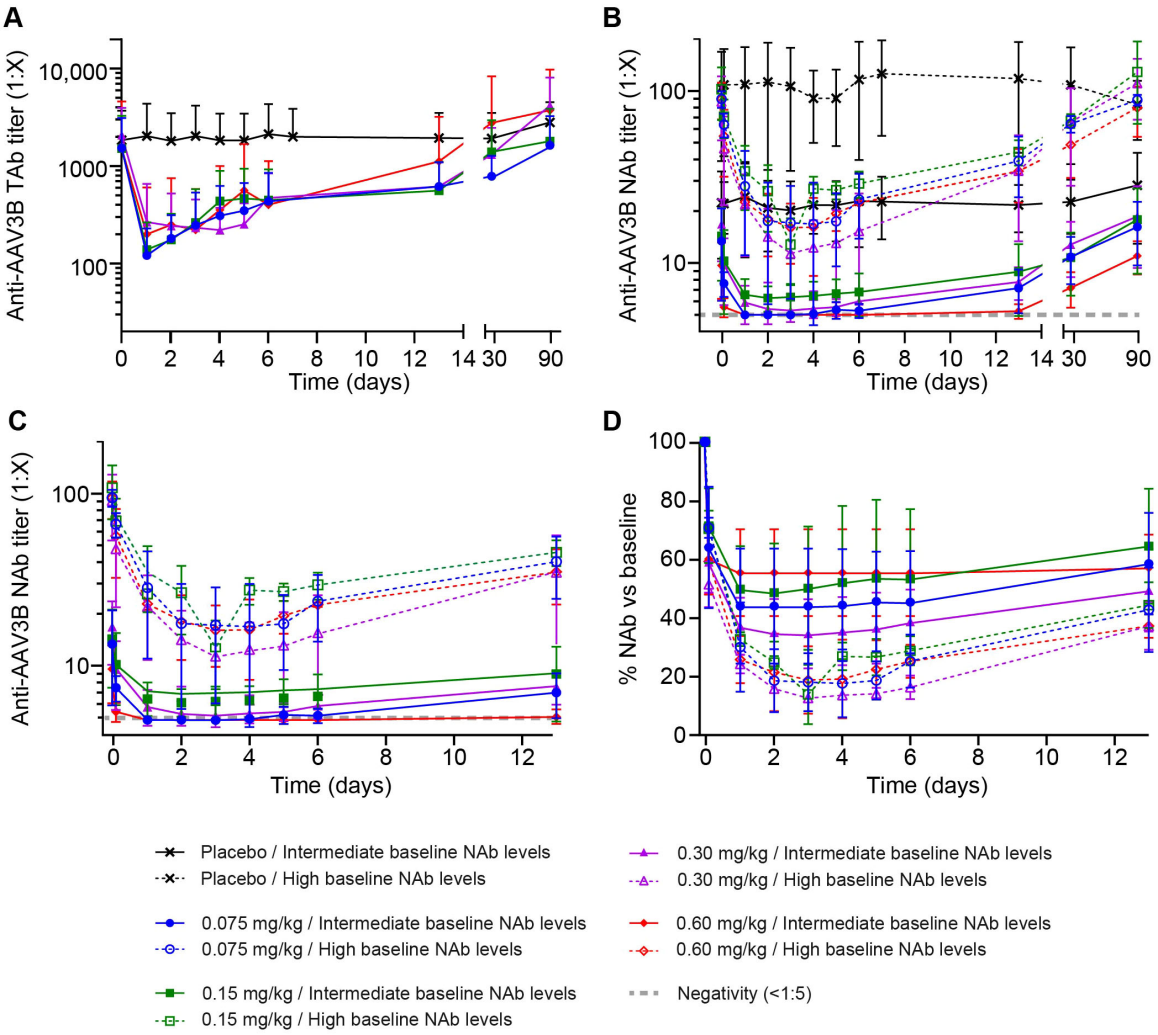
Pharmacodynamic analysis of the effect of VTX-PID on anti-AAV3B TAb and anti-AAV3B NAb levels over time. **(A)** TAbs up to Day 90 post dose, **(B)** NAbs up to Day 90 post dose, **(C)** focus on NAbs up to 13 days post dose, **(D)** results of NAbs in % (normalized with NAbs baseline) up to 13 days post dose. The dashed gray line represents the negativity threshold at <1:5. For NAbs only: solid and dashed lines and closed and open symbols represent the subpopulations with intermediate or high baseline NAb levels respectively. Means ± standard deviation are represented. AAV3B, adeno-associated virus serotype 3B; NAb, neutralizing antibody; TAb, total antibody.

The original version of this article has been updated.

In the **Acknowledgments**, the mention of “writing assistance provided by Nicolisha Narainpersad, PhD, of Parexel” was omitted in the final published version. The correct **Acknowledgments** statement is: “The authors would like to thank the participants who participated in the study and the investigators and clinical research staff from the study center. Writing assistance was provided by Nicolisha Narainpersad, PhD, of Parexel”.

The original version of this article has been updated.

