# Supplementary material for: VTX-PID as a novel recombinant immunoglobulin G–degrading enzyme (IdeS) for efficient AAV-based gene therapy in participants with neutralizing antibodies: results of the phase I first-in-human NAVIgATE study
Source: Front Immunol. 2026 May 20;17:1824802. doi: 10.3389/fimmu.2026.1824802 (PMC13231502; doi:10.3389/fimmu.2026.1824802)
Supplement: Supplementary file 1 [file Table1.docx]

Supplementary Material

# Supplementary Figure

**Supplementary Figure 1.** **(A)** Phase I first-in-human double-blind, randomized study design and **(B)** dose escalation scheme. AAV3B, adeno-associated virus serotype 3B; NAb, neutralizing antibodies; SRC, safety review committee.


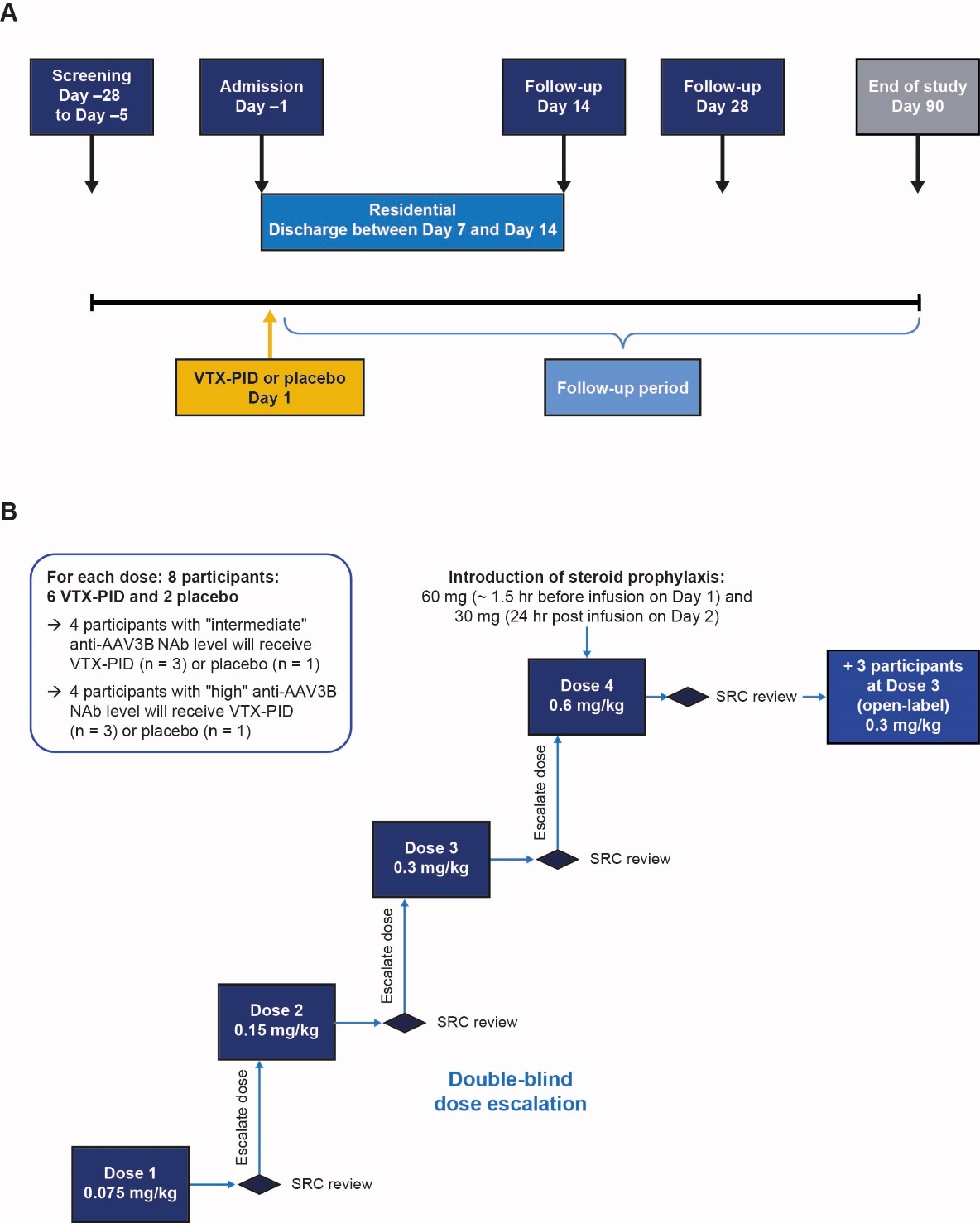


# Supplementary Tables

**Table S1. Safety summary across the four cohorts and pooled placebo group.**

| **Participants with event, n (%)** | **Cohort 1**  **VTX-PID**  **(0.075 mg/kg)**  **(*n*=6)** | **Cohort 2**  **VTX-PID**  **(0.15 mg/kg)**  **(*n*=6)** | **Cohort 3**  **VTX-PID**  **(0.3 mg/kg)**  **(*n*=9)*** | | | **Cohort 4**  **VTX-PID**  **(0.6 mg/kg)**  **(*n*=6)** | | | **Pooled**  **placebo**  **(*n*=8)** | **Overall**  **(*n*=35)*** |
| --- | --- | --- | --- | --- | --- | --- | --- | --- | --- | --- |
|  |  |  | **Withoutcortico-steroid** | **With cortico-steroid** | **Overall** | **Without cortico-steroid** | **With cortico-steroid** | **Overall** |  |  |
| ≥1 AE | 6 (100) | 3 (50.0) | 5 (55.6) | 3 (33.3) | 8 (88.9) | 4 (66.7) | 2 (33.3) | 6 (100) | 4 (50.0) | 27 (77.1) |
| ≥1 serious AE | 0 | 0 | 0 | 0 | 0 | 0 | 0 | 0 | 0 | 0 |
| ≥1 TEAE | 6 (100) | 3 (50.0) | 5 (55.6) | 3 (33.3) | 8 (88.9) | 4 (66.7) | 2 (33.3) | 6 (100) | 4 (50.0) | 27 (77.1) |
| ≥1 serious TEAE | 0 | 0 | 0 | 0 | 0 | 0 | 0 | 0 | 0 | 0 |
| TEAE severity |  |  |  |  |  |  |  |  |  |  |
| Overall | 6 (100) | 3 (50.0) | 5 (55.6) | 3 (33.3) | 8 (88.9) | 4 (66.7) | 2 (33.3) | 6 (100) | 4 (50.0) | 27 (77.1) |
| Mild | 5 (83.3) | 2 (33.3) | 5 (55.6) | 3 (33.3) | 8 (88.8) | 4 (66.7) | 2 (33.3) | 6 (100) | 4 (50.0) | 25 (71.4) |
| Moderate | 3 (50.0) | 3 (50.0) | 4 (44.4) | 3 (33.3) | 7 (77.8) | 2 (33.3) | 2 (33.3) | 4 (66.7) | 0 | 17 (48.6) |
| Severe | 0 | 0 | 1 (11.1) | 0 | 1 (11.1) | 0 | 1 (16.7) | 1 (16.7) | 0 | 2 (5.7) |
| TEAE relationship to study drug |  |  |  |  |  |  |  |  |  |  |
| Overall | 6 (100) | 3 (50.0) | 5 (55.6) | 3 (33.3) | 8 (88.9) | 4 (66.7) | 2 (3.33) | 6 (100) | 4 (50.0) | 27 (77.1) |
| Related^†^ | 3 (50.0) | 3 (50.0) | 4 (44.4) | 3 (33.3) | 7 (77.8) | 4 (66.7) | 2 (3.33) | 6 (100) | 1 (12.5) | 20 (57.1) |
| Not related | 5 (83.3) | 1 (16.7) | 4 (44.4) | 2 (22.2) | 6 (66.7) | 2 (33.3) | 2 (3.33) | 4 (66.7) | 4 (50.0) | 20 (57.1) |
| TEAE led to study drug discontinuation | 0 | 0 | 0 | 0 | 0 | 0 | 0 | 0 | 0 | 0 |

*Three nonrandomized participants were included and treated with 0.3 mg/kg VTX-PID (Cohort 3) after the end of the escalation phase (Day 14 of the last participant for Cohort 4) along with randomized participants. ^†^Related TEAEs includes both “TEAE only related to study drug” and “TEAE related to study drug combined with other trial drug.” AE, adverse event; TEAE, treatment-emergent adverse event.

**Table S2.** **TEAEs by severity across the four cohorts, pooled placebo group, and overall.**

| **Participants with event, n (%)** | **Severity** | **Cohort 1**  **VTX-PID**  **(0.075 mg/kg)**  **(*n*=6)** | **Cohort 2**  **VTX-PID**  **(0.15 mg/kg)**  **(*n*=6)** | **Cohort 3**  **VTX-PID**  **(0.3 mg/kg)**  **(*n*=9)*** | | | **Cohort 4**  **VTX-PID**  **(0.6 mg/kg)**  **(*n*=6)** | | | **Pooled**  **placebo**  **(*n*=8)** |
| --- | --- | --- | --- | --- | --- | --- | --- | --- | --- | --- |
|  |  |  |  | **Withoutcortico-steroid** | **With cortico-steroid** | **Overall** | **Without cortico-steroid** | **With cortico-steroid** | **Overall** |  |
| Any TEAE | Overall | 6 (100) | 3 (50.0) | 5 (55.6) | 3 (33.3) | 8 (88.9) | 4 (66.7) | 2 (3.33) | 6 (100) | 4 (50.0) |
|  | Mild | 3 (50.0) | 0 | 1 (11.1) | 0 | 1 (11.1) | 2 (33.3) | 0 | 2 (33.3) | 4 (50.0) |
|  | Moderate | 3 (50.0) | 3 (50.0) | 3 (33.3) | 3 (33.3) | 6 (66.7) | 2 (33.3) | 1 (16.7) | 3 (50.0) | 0 |
|  | Severe | 0 | 0 | 1 (11.1) | 0 | 1 (11.1) | 0 | 1 (16.7) | 1 (16.7) | 0 |
| Most frequent TEAEs^†^ |  |  |  |  |  |  |  |  |  |  |
| Myalgia | Mild | 2 (33.3) | 0 | 1 (11.1) | 1 (11.1) | 2 (22.2) | 2 (33.3) | 0 | 2 (33.3) | 0 |
|  | Moderate | 1 (16.7) | 2 (33.3) | 1 (11.1) | 1 (11.1) | 2 (22.2) | 0 | 2 (33.3) | 2 (33.3) | 0 |
|  | Severe | 0 | 0 | 0 | 0 | 0 | 0 | 0 | 0 | 0 |
| Arthralgia | Mild | 0 | 0 | 1 (11.1) | 2 (22.2) | 3 (33.3) | 1 (16.7) | 0 | 1 (16.7) | 0 |
|  | Moderate | 0 | 1 (16.7) | 1 (11.1) | 0 | 1 (11.1) | 1 (16.7) | 1 (16.7) | 2 (33.3) | 0 |
|  | Severe | 0 | 0 | 1 (11.1) | 0 | 1 (11.1) | 0 | 0 | 0 | 0 |
| Headache | Mild | 2 (33.3) | 0 | 1 (11.1) | 0 | 1 (11.1) | 0 | 2 (33.3) | 2 (33.3) | 1 (12.5) |
|  | Moderate | 0 | 0 | 0 | 1 (11.1) | 1 (11.1) | 0 | 0 | 0 | 0 |
|  | Severe | 0 | 0 | 0 | 0 | 0 | 0 | 0 | 0 | 0 |
| C-reactive protein  increased | Mild | 0 | 0 | 0 | 0 | 0 | 0 | 0 | 0 | 0 |
|  | Moderate | 0 | 0 | 3 (33.3) | 0 | 3 (33.3) | 2 (33.3) | 0 | 2 (33.3) | 0 |
|  | Severe | 0 | 0 | 0 | 0 | 0 | 0 | 1 (16.7) | 1 (16.7) | 0 |
| Pyrexia | Mild | 0 | 0 | 3 (33.3) | 0 | 3 (33.3) | 1 (16.7) | 1 (16.7) | 2 (33.3) | 0 |
|  | Moderate | 0 | 0 | 0 | 0 | 0 | 1 (16.7) | 0 | 1 (16.7) | 0 |
|  | Severe | 0 | 0 | 0 | 0 | 0 | 0 | 0 | 0 | 0 |
| Abdominal discomfort | Mild | 0 | 0 | 2 (22.2) | 0 | 2 (22.2) | 1 (16.7) | 1 (16.7) | 2 (33.3) | 0 |
|  | Moderate | 0 | 0 | 0 | 0 | 0 | 0 | 0 | 0 | 0 |
|  | Severe | 0 | 0 | 0 | 0 | 0 | 0 | 0 | 0 | 0 |
| Infusion-related reaction | Mild | 0 | 0 | 0 | 0 | 0 | 1 (16.7) | 0 | 1 (16.7) | 0 |
|  | Moderate | 0 | 0 | 0 | 1 (11.1) | 1 (11.1) | 2 (33.3) | 0 | 2 (33.3) | 0 |
|  | Severe | 0 | 0 | 0 | 0 | 0 | 0 | 0 | 0 | 0 |
| Fatigue | Mild | 0 | 0 | 0 | 1 (11.1) | 1 (11.1) | 0 | 1 (16.7) | 1 (16.7) | 1 (12.5) |
|  | Moderate | 0 | 0 | 0 | 0 | 0 | 0 | 0 | 0 | 0 |
|  | Severe | 0 | 0 | 0 | 0 | 0 | 0 | 0 | 0 | 0 |
| Lymphadeno-pathy | Mild | 0 | 0 | 0 | 1 (11.1) | 1 (11.1) | 1 (16.7) | 0 | 1 (16.7) | 0 |
|  | Moderate | 0 | 0 | 0 | 0 | 0 | 1 (16.7) | 0 | 1 (16.7) | 0 |
|  | Severe | 0 | 0 | 0 | 0 | 0 | 0 | 0 | 0 | 0 |
| Nasopharyn-gitis | Mild | 0 | 1 (16.7) | 0 | 0 | 0 | 0 | 0 | 0 | 2 (25.0) |
|  | Moderate | 0 | 0 | 0 | 0 | 0 | 0 | 0 | 0 | 0 |
|  | Severe | 0 | 0 | 0 | 0 | 0 | 0 | 0 | 0 | 0 |
| Rash | Mild | 1 (16.7) | 0 | 0 | 0 | 0 | 0 | 0 | 0 | 0 |
|  | Moderate | 0 | 0 | 1 (11.1) | 0 | 1 (11.1) | 1 (16.7) | 0 | 1 (16.7) | 0 |
|  | Severe | 0 | 0 | 0 | 0 | 0 | 0 | 0 | 0 | 0 |

*Three nonrandomized participants were included and treated with 0.3 mg/kg VTX-PID (Cohort 3) after the end of the escalation phase (Day 14 of the last participant for Cohort 4) along with randomized participants. ^†^TEAEs of any severity by preferred term are listed if they were reported in ≥3 participants overall. TEAE, treatment-emergent adverse event.

**Table S3.** **Summary statistics of VTX‑PID plasma pharmacokinetic parameters by cohort or dose levels and strata based on baseline antidrug antibody (ADA) titer categories (pharmacokinetic analysis set).**

| **PK parameter** | **Statistic** | **Cohort 1**  **VTX-PID**  **(0.075 mg/kg)**  **(*n*=6)** | | **Cohort 2**  **VTX-PID**  **(0.15 mg/kg)**  **(*n*=6)** | | **Cohort 3**  **VTX-PID**  **(0.3 mg/kg)**  **(*n*=9)*** | | **Cohort 4**  **VTX-PID**  **(0.6 mg/kg)**  **(*n*=6)** | |
| --- | --- | --- | --- | --- | --- | --- | --- | --- | --- |
|  |  | **Titer ≤1:1000** | **Titer >1:1000** | **Titer ≤1:1000** | **Titer >1:1000** | **Titer ≤1:1000** | **Titer >1:1000** | **Titer ≤1:1000** | **Titer >1:1000** |
| C_max_, μg/mL | n | 5 | 1 | 4 | 2 | 4 | 5 | 1 | 5 |
|  | Arithmetic mean (SD) | 1.78 (0.24) | NC | 3.49 (0.40) | NC | 6.53 (1.04) | 7.76 (1.09) | NC | 13.92 (1.70) |
| t_max_, h | n | 5 | 1 | 4 | 2 | 4 | 5 | 1 | 5 |
|  | Median (range) | 0.75  (0.74-0.75) | NC  (1.51-1.51) | 0.78  (0.51-1.05) | NC  (0.75-1.49) | 0.63  (0.50-0.75) | 0.75  (0.50-1.01) | NC  (1.01-1.01) | 1.25  (1.00-1.35) |
| DNC_max_, kg*μg/mL/mg | n | 5 | 1 | 4 | 2 | 4 | 5 | 1 | 5 |
|  | Arithmetic mean (SD) | 22.25 (3.05) | NC | 23.27 (2.69) | NC | 20.90 (3.26) | 25.53 (3.35) | NC | 22.94 (2.57) |
| AUC_last_, h*μg/mL | n | 5 | 1 | 4 | 2 | 4 | 5 | 1 | 5 |
|  | Arithmetic mean (SD) | 5.42 (1.85) | NC | 22.45 (14.86) | NC | 24.77 (3.44) | 146.6 (85.59) | NC | 188.6 (63.82) |
| AUC_inf_, h*μg/mL | n | 0 | 0 | 2 | 1 | 4 | 3 | 1 | 0 |
|  | Arithmetic mean (SD) | NC | NC | NC | NC | 29.52 (4.00) | 201.6 (91.15) | NC | NC |
| t_½_, h | n | 5 | 1 | 2 | 2 | 4 | 4 | 1 | 3 |
|  | Arithmetic mean (SD) | 3.20 (1.11) | NC | NC | NC | 2.94 (0.39) | 54.78 (10.79) | NC | 116.9 (16.24) |
|  | Geometric mean (geometric CV%) | 3.08 (30.6) | NC | NC | NC | 2.92 (13.7) | 54.00 (19.7) | NC | 116.2 (13.8) |
| CL, mL/h/kg | n | 0 | 0 | 2 | 1 | 4 | 3 | 1 | 0 |
|  | Arithmetic mean (SD) | NC | NC | NC | NC | 10.75 (1.59) | 1.72 (0.69) | NC | NC |
| V_z_, mL/kg | n | 0 | 0 | 2 | 1 | 4 | 3 | 1 | 0 |
|  | Arithmetic mean (SD) | NC | NC | NC | NC | 45.33 (8.13) | 121.4 (42.32) | NC | NC |

*Three nonrandomized participants were included and treated with 0.3 mg/kg VTX-PID (Cohort 3) after the end of the escalation phase (Day 14 of the last participant for Cohort 4) along with randomized participants. AUC_inf_, AUC from time zero extrapolated to infinity; AUC_last_, AUC from time zero to the last quantifiable concentration; C_max_, maximum observed concentration; CL, apparent clearance following intravascular administration; CV, coefficient of variation; DNC_max_, dose‑normalized C_max_; NC, not calculable; SD, standard deviation; t_½_, apparent terminal elimination half-life; t_max_, time corresponding to occurrence of C_max_; V_z_, apparent volume of distribution during terminal phase following intravascular dosing.

**Table S4. Summary statistics of the duration of negative anti-AAV3B NAb status** **across the four cohorts, pooled placebo group, overall, and strata based on screening anti-AAV3B NAb levels (pharmacodynamic analysis set).**

| **Category statistics** | **Cohort 1**  **VTX-PID**  **(0.075 mg/kg)** | **Cohort 2**  **VTX-PID**  **(0.15 mg/kg)** | **Cohort 3**  **VTX-PID**  **(0.3 mg/kg)** | **Cohort 4**  **VTX-PID**  **(0.6 mg/kg)** | **Pooled**  **placebo**  **(*n*=8)** |
| --- | --- | --- | --- | --- | --- |
|  | **Intermediate (*n*=3)** | **Intermediate (*n*=4)** | **Intermediate (*n*=6)*** | **Intermediate (*n*=4)** |  |
| Duration, hours, n (%) |  |  |  |  |  |
| 0 | 0 | 3 (75.0) | 2 (33.3) | 0 | 8 (100) |
| >0 and ≤24 | 0 | 0 | 1 (16.7) | 0 | 0 |
| >24 and ≤48 | 1 (33.3) | 0 | 1 (16.7) | 0 | 0 |
| >48 and ≤72 | 0 | 0 | 0 | 0 | 0 |
| >72 and ≤96 | 0 | 0 | 0 | 0 | 0 |
| >96 and ≤120 | 2 (66.7) | 0 | 1 (16.7) | 0 | 0 |
| >120 and ≤144 | 0 | 0 | 0 | 0 | 0 |
| >144 | 0 | 1 (25.0) | 1 (16.7) | 4 (100) | 0 |

*Three nonrandomized participants were included and treated with 0.3 mg/kg VTX-PID (Cohort 3) after the end of the escalation phase (Day 14 of the last participant for Cohort 4) along with randomized participants. AAV3B, adeno-associated virus serotype 3B; NAbs, neutralizing antibodies.
